# Knowledge gaps of STIs in Africa; Systematic review

**DOI:** 10.1371/journal.pone.0213224

**Published:** 2019-09-12

**Authors:** Marwan M. Badawi, Maryam A. SalahEldin, Alaa B. Idris, Elfatih A. Hasabo, Zeinab H. Osman, Widad M. Osman

**Affiliations:** 1 Medical Unit, Higher Academy for Strategic and Security Studies, Alamal Hospital, Khartoum, Sudan; 2 Medical Microbiology Department, Faculty of Medical Laboratory Sciences, University of Khartoum, Khartoum, Sudan; 3 Faculty of Medicine, University of Bahri, Khartoum, Sudan; 4 Faculty of Medicine, University of Khartoum, Khartoum, Sudan; 5 Faculty of Medical Laboratory Sciences, Sudan International University, Khartoum, Sudan; 6 Faculty of Medicine, National Ribat University, Khartoum, Sudan; Translational Health Science & Technology Institute, INDIA

## Abstract

Sexually Transmitted Infections (STIs) are ambiguous burden of tremendous health, social and economic consequences. The current systematic review was conducted in order to determine awareness and knowledge of Africans toward sexually transmitted infections, not only concerning HIV/AIDS, but also other STIs such as gonorrhea, syphilis, HBV, HCV and HPV. A systematic review of literature was conducted, studies were retrieved and selected after fulfilling the inclusion criteria as well as passing the assessment procedure. Related data was extracted, quantitative analysis was conducted among participants who responded to questions related to HIV, HBV, HCV, HPV or STIs knowledge, sensitivity analysis as well as subgroup analysis were also conducted. Seventy four articles addressing knowledge among 35 African countries were included and 136 questions were analyzed and synthesized. The question “does using condom reduces HIV transmission?” was answered by 1,316,873 Africans in 35 countries, 66.8% [95% Cl; 62.6, 70.9] answered yes. While the question “is sexual contact a possible route of HBV transmission?” was answered by 7,490 participants in 5 countries; 42.5% [95% Cl; 20.4, 64.7] answered yes. The differences observed among populations are highlighting the possibility for improvement by directing light toward specific populations as well as addressing specific awareness knowledge to ensure that the general as well as the related specific preventive knowledge is improved.

## Introduction

Sexually transmitted Infections (STIs) are ambiguous burden of tremendous health, social and economic consequences. Many STIs are hidden because many people may feel stigmatized when addressing them. Moreover, the committee on prevention and control of sexually transmitted diseases in USA estimated that the annual costs of selected major STDs are approximately $10 billion or, if sexually transmitted HIV is included, $17 billion [1].

According to UNAIDS; almost 37 million people globally were living with HIV in 2017, sub-Saharan Africa accounted for 66% of the cases, 68% of new adult HIV infections, 92% of new infections in children and 72% of all AIDS-related deaths. Earlier in 2009, Swaziland topped the world’s HIV epidemic countries with a 26% prevalence among adults, while South Africa was the country with the world’s largest prevalence of people living with HIV as 5.6 million [2,3].

On the other hand and according to WHO; an estimated 257 million people are living with HBV infection with the highest prevalence in the Western Pacific Region and the African Region as 6.2% and 6.1% of the adult population are infected, respectively. About 1% of persons living with HBV infection (2.7 million people) are also infected with HIV. Moreover, approximately 399,000 people die each year from hepatitis C infection. Furthermore, the estimated global HPV prevalence is 11.7% with the Sub-Saharan Africa having the largest burden as well(24.0%) [4–6].

Africa is considered the continent with the lowest Gross Domestic Product (GDP) as most African countries fall within the lower-middle to low income countries classification. In March 2013, despite of the predicted uprising in African economy in the following decades, Africa was identified as the world’s poorest inhabited continent; Africa’s entire combined GDP is estimated to be barely a third of the United States’, this could straightforwardly influencescreening opportunities, medical consultations as well as treatment options. Taking that under consideration; a strategyfor STIs containment in Africa should primarily emphasize prevention and its related knowledge. Chan and Tsai in their study represented STIs related awareness levels based on data collected from 33 sub-Saharan African countries. Although their study determined the estimated awareness according to data collected from 2003 to 2015 as well as a knowledge trend among each participated country was illustrated, awareness of five questions were assessed regarding HIV only. The current systematic review was conducted in order to determine awareness and knowledge of Africans of sexually transmitted infections, not only concerning HIV/AIDS, but also other STIs such as, gonorrhea, syphilis, HBV, HCV and HPV and concerning all awareness determinants that are reported in the literature [7,8].

## Materials and methods

### Search strategy

To identify relevant studies; a systematic review of the literature was conducted in the 1^st^ of December 2018. The review was regulated in accordance with the PRISMA (Preferred Reporting Items for Systematic Reviews and Meta-Analyses) Statement [9] (S1 Table). A comprehensive search was operated in PubMed, Embase, Google scholar, Scopus, Index Copernicus, DOAJ, EBSCO-CINAHL, Cochrane databases without language limits (studies written in French were later excluded). To obtain a current situation evidence; only studies published in or after 2010 were included. Furthermore, all studies where the data collection process took place before 2010 were also excluded, the only exception was if the collection process started before 2010 and ended in 2010 or afterwards. The keywords used in PubMed was as follow:

((HIV[Tiab] OR syphilis[Tiab] OR gonorrhea[Tiab] OR sexual behavior[Tiab] OR “men who have sex with men”[Tiab] OR condom[Tiab] OR “herpes simplex virus”[Tiab] OR “sex workers”[Tiab] OR sex [tiab]OR human immunodeficiency virus[Tiab] OR HBV[Tiab] OR HCV[Tiab] OR HPV[Tiab] OR prostitutes[Tiab]) AND (behavior [Ti] OR risk [ti] OR awareness[Ti] OR knowledge[Ti] OR assessment[Ti]) AND (africa[Tiab] OR algeria[Tiab] OR angola[Tiab] OR benin[Tiab] OR botswana[Tiab] OR burkinafaso[Tiab] OR burundi[Tiab] OR caboverde[Tiab] OR cameroon[Tiab] OR central african republic[Tiab] OR CAR[Tiab] OR chad[Tiab] OR comoros[Tiab] OR “democratic republic of the congo”[Tiab] OR “republic of the congo”[Tiab] OR cote d’ivoire[Tiab] OR djibouti[Tiab] OR egypt[Tiab] OR equatorial guinea[Tiab] OR eritrea[Tiab] OR eswatini[Tiab] OR swaziland[Tiab] OR ethiopia[Tiab] OR gabon[Tiab] OR gambia[Tiab] OR ghana[Tiab] OR guinea[Tiab] OR guinea-bissau[Tiab] OR kenya[Tiab] OR lesotho[Tiab] OR liberia[Tiab] OR libya[Tiab] OR madagascar[Tiab] OR malawi[Tiab] OR mali[Tiab] OR mauritania[Tiab] OR mauritius[Tiab] OR morocco[Tiab] OR mozambique[Tiab] OR namibia[Tiab] OR niger[Tiab] OR nigeria[Tiab] OR rwanda[Tiab] OR (sao tome principe[Tiab] OR senegal[Tiab] OR seychelles[Tiab] OR sierra leone[Tiab] OR somalia[Tiab] OR south africa[Tiab] OR south sudan[Tiab] OR sudan[Tiab] OR swaziland[Tiab] OR eswatini[Tiab] OR tanzania[Tiab] OR togo[Tiab] OR tunisia[Tiab] OR uganda[Tiab] OR zambia[Tiab] OR zimbabwe[Tiab])).

Moreover, to optimize our search, hand searches of reference lists of included articles were also performed.

#### Study selection and data extraction

All authors independently assessed titles and abstracts for eligibility, and any disagreement was resolved through discussion. A copy of the full text was obtained for all research articles that were available and approved in principle to be included. Abstraction of data was in accordance with the task separation method; method and result sections in each study were separately abstracted in different occasions to reduce bias. Moreover, data abstraction was conducted with no consideration of author’s qualifications or expertise as described in details previously [10]. Studies assessed the knowledge of parasitic infections as well as studies conducted among healthcare workers (clinicians, laboratory specialists, nurses, dentists and midwives) were excluded. If a data regarding the period of conduction is missing; the reference list was crossed, if any cited study wasfound to bepublished after 2010;authors of the current review agreed to predict that the study is conducted after 2010 and hence it was considered for inclusion, and it was designed to be addressed later in the review as (conducted after 2010), otherwise the study was excluded. All studies measuring awareness level with scores or if it is generally good or moderate or poor without determining further details were also excluded. Each research article was screened for all relevant information and recorded in the data extraction file (Microsoft Excel), as one article may report outcome of awareness and/or knowledge and/or attitude toward specific sexually transmitted infection or toward several STIs, in a single population or among several ones. Moreover, data from each method section was extracted using a predefined set of variables; study characteristics, type of participants, study population size, geographical region and the period of the study conduction.

#### Assessment of quality

Each included article was evaluated based on a framework for making a summary assessment of the quality. The related published literature was crossed, then a framework was structured specifically to determine the level of representativeness of the studied population and to judge the strength of the estimates provided. Six questions were to be answered in each article, each answer represent either 1 score for yes, 0 score for No or 0 score for not available; a total score for risk of bias and quality was calculated by adding up the scores in all six domains, resulting in a score of between 0 and 6. The highest score indicates the highest quality, studies with a score for quality greater or equal to 3 (higher quality) were included in the review.

The six domains were: is the study objective clearly defined?, is the study sample completely determined?, is the study population clearly defined and specified?, is the response rate of participants above 70%?, is the methodology rigorous? and is the data analysis rigorous?

Trim and Fill method was used to assess the risk of publication bias in each question responses in the included studies [11]. Publication bias was assessed separately for each question-corresponding responses only if the question was addressed and answered in studies equal or greater than ten.

#### Quantitative analysis

Meta-analysis was performed—whenever possible using Review Manager Software (Version 5.3). In studies where the Standard Error (SE) is not reported; the following formula was used to calculate it: SE = √p (1-p)/ n where p stands for Prevalence. The software automatically provided the Confidence Interval (CI) according to the calculated SE, if the CI is provided in a study; it was introduced accordingly. The heterogeneity of each meta-analysis was assessed as described in details previously [10], the random effect was favored over the fixed effect model in all meta-analysis established as variations between studies is predicted to be probable due to the diversity of the study populations. Sensitivity analysis was also approached to determine the effect of studies conducted in populations proposed to behave in indifference manners or proposed to be more aware on the overall pooled prevalence. Moreover, subgroup analysis was also conducted -whenever suitable to determine awareness level in specific country or population. A question to take part in the meta-analysis has to be included in at least two studies. Moreover, for providing a better image as well as emphasizing potential research gaps; all HIV-related questions that are proposed to be of interest according to the objective of the current review, and was answered by at least 1,000 Africans, but included only in one study, were also provided alongside their related references. Nevertheless, questions related to other STIs were provided regardless of the number of participants due to their minority. Questions with similar outcome were proposed to be the same (e.g: the question “do you think sexual intercourse will increase the risk of HIV transmission?” and the question “is HIV sexually transmitted?” were considered as one question).

## Results

### Studies included

**Fig 1:**
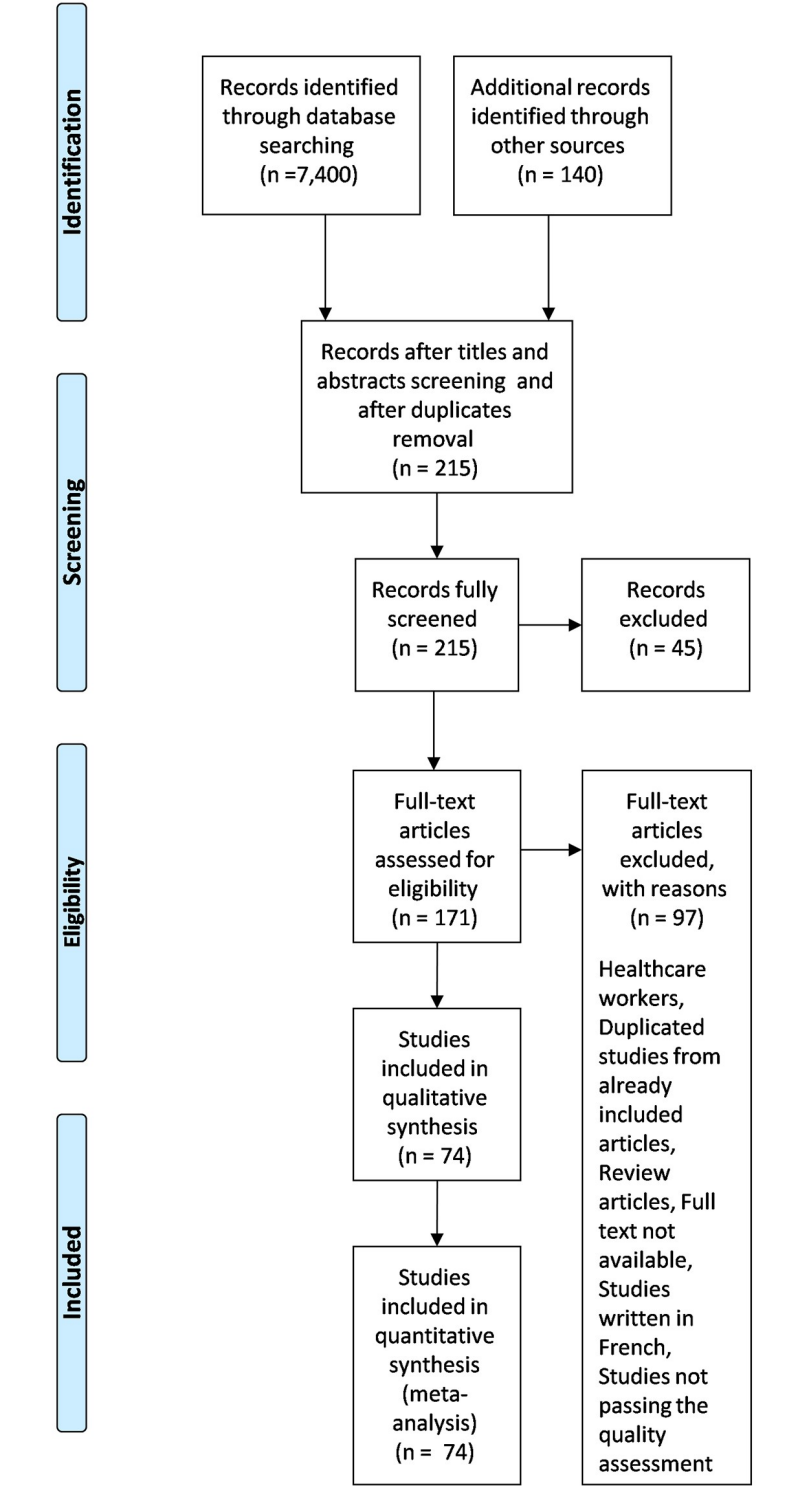
Literature search and selection of studies (PRISMA flow diagram).

A total of 7,540 articles were identified from the search strategy including hand searches of reference lists of included original research articles and reviews. From these, 7,453 articles were excluded. Seventy four articles met our inclusion criteria and passed the quality assessment procedure [8,12–84]. The articles reported specific awareness determinants and/or knowledge and/or attitudes of an African population regarding STIs as general and/or HBV and/or HCV and/or HPV and/or HIV. (Fig 1) illustrates the PRISMA flow diagram. The included articles are depicted in (Table 1). Assessment of the quality of included studies is depicted in (S2 Table).

**Table 1:** Characteristics of included studies.

Study	Year of publication	Year/s of conduction	City/Region/Country	study population/s	Assessment of knowledge of	sample size	Gender	Participants’ Age
**Abdulai *et al*** [12]	2016	2010	Kintampo North/Brong Ahafo Region/Ghana	Pregnant women	HBV	504	Female	20–32
**Abimanyi-Ochom *et al***[13]	2017	2011	Uganda	General population	HIV	10,969	Both	15–49
**Aderemi *et al***[14]	2013	After 2010	Oyo State/Nigeria	Students	HIV	600	Both	12–19
**Adoba *et al***[15]	2015	2015	Obuasi/Ghana	Barbers	HBV/HCV	200	Male	20–29
**Ajide and Balogun**[16]	2018	After 2010	Ibadan/Nigeria	Students	HIV	240	Both	15–17
**Akokuwebe *et al***[17]	2016	After 2010	Ikeji-Arakeji/Osun/Nigeria	Adolescents	HIV/STIs	341	Both	14–18
***Amu and Adegun***[18]	2015	After 2010	Ado Ekiti/Nigeria	Students	HIV/STIs	540	Both	10–14
***Appiah-Agyekum et al***[19]	2013	After 2010	Accra/Ghana	Students	HIV	260	Female	16- ≥19
***Aroke et al***[20]	2018	2016	Centre Region, North Region and South Region/Cameroon	Medical students	HBV	714	Both	21–23
***Asefa and Beyene***[21]	2013	2012	Southern Nations, Nationalities, and Peoples’ Region/ Ethiopia	Pregnant women	HIV	1,325	Female	15–49
***Audet et al***[22]	2012	After 2010	Zambézia/Mozambique	General population	HIV	349	Both	Median 32
***Azodo et al***[23]	2014	2010	Enugu/Nigeria	Dental tech students	HIV	198	Both	20- ≥27
***Becker et al***[24]	2015	2009–2010	Durban/KwaZulu-Natal/South Africa	General population	HIV	2,477	Both	N.A
***Carlos et al***[25]	2015	2010	Kinshasa/ Democratic Republic of Congo	Outpatients and blood donors	HIV	1,614	Both	15–49
***Chan and Tsai***[8]	2018	2003–2015	33 Countries	General population	HIV	1,187,077	Both	N.A
**Chaquisse et al**[26]	2018	2013–2014	Nampula/Mozambique	Pregnant women	HIV/HBV/HCV	1,186	Female	median 22
**Chard *et al***[27]	2017	After 2010	South Africa	men who indicated an interest in men	HIV	386	Male	mean 33
**Cheng *et al***[28]	2015	2013–2014	Kumasi/Ghana	pregnant women	HBV	209	Female	N.A
**Chimoyi *et al***[29]	2015	2013	Gohannesburg/Gauteng/South africa	General population	HIV	1146	Both	N.A
**Ciampa *et al***[30]	2012	2011	Zambezia/Mozambique	prenatal care in women	HIV	348	Female	median 24
**Darteh *et al***[31]	2016	2011	KwesimintsimZongo/Ghana	adolescents, general population	HIV	902	Both	mean 14
**Demsiss *et al***[32]	2018	2017	South Wollo Zone/Amhara/Ethiopia	medicine and health science students	HBV/HCV	408	Both	mean 26 ± 4
**Elbadawi *et al***[33]	2016	2016	Khartoum/Sudan	university students	HIV	556	Both	N.A
**Engelbrecht *et al***[34]	2017	2012	Moretele Sub-district/Bojanala District/North West province/South Africa	home-based carers	HIV	144	Both	median 35
**Eni *et al***[35]	2018	2016	Lagos, Ogun and Abia/Nigeria	University students, general population	HPV	758	Both	16–39
**Ezenwa *et al***[36]	2013	2012	Shomolu Local Government Area/Lagos/Nigeria	mothers of female adolescents	HPV	290	Female	24–62
**Faleye *et al***[37]	2014	2012–2013	Durban/KwaZulu- Natal/South africa	Male medical circumcision clients	HIV	394	Male	mean 28±9
**Faust *et al***[38]	2017	2013	Nigeria	general population	HIV	56,307	Both	15–49
**Faye *et al***[39]	2013	May, 2010	Senegal	seafarers	HIV	400	Male	28–48
**Frambo *et al***[40]	2014	2012	Buea Health District/Cameroon	pregnant women	HBV	176	Female	17–42
**Funmilayo *et al***[41]	2018	2014	Lagos/Nigeria	university students	HPV	280	Both	15–40
**George *et al***[42]	2013	2010	Northern Cape province/South Africa	rural based traditional healers	HIV	186	Both	N.A
**Griffith *et al***[43]	2015	2013	Masiphumelele/Cape Town/South Africa	HIV-Infected and HIV-Uninfected Adolescent Women	HPV	30	Female	16–21
***Jodaetal***[44]	2013	After 2010	Lagos/Nigeria	students	Gonorrhoea/STIs	30	Both	15–35
***Kiderlenetal***[45]	2015	2011	Namibia	employees in truck transport sector	HIV	483	Both	≤19-≥60
***Kufaetal***[46]	2018	After 2010	Eastern Cape,WesternCape,FreeState,Gauteng/South Africa	STI service attendees	HIV	1,054	Both	23–32
***Laraquietal***[47]	2017	2014	Morocco	seafarers	STI,HIV	1447	Male	28–44
**Lawan *etal***[48]	2012	2011	Kano/Nigeria	FSW	HIV	124	Females	24–28
**Makwe *etal***[49]	2012	2010	Lagos/Nigeria	students	HPV	368	Females	16–29
**Mason *etal***[50]	2013	after 2010	Gambia	Men Who Have Sex with Men	HIV	207	Males	≥16
**Massey *etal***[51]	2017	2014	Dakar, Thies, Fatick, Mbour and Ziguinchor/Senegal	Adolescents and young adults	HPV	2,286	Both	14–22
**Mesfin *etal***[52]	2013	2013	Harar town/Harari/Ethiopia	Students	HBV	322	Both	20–24
**Mkumbo *etal***[53]	2013	after 2011	Dar es Salaam, Morogoro and Tanga/Tanzania	Students	HIV	362	Both	Mean 25
**Mouallif *etal***[54]	2014	2012	Casablanca, Marrakech, Chaouiya-Ouardiguaand Tangier/Morocco	parents	HPV	852	Both	19–67
**Nabukenya *etal***[55]	2018	2011	Uganda	older adults	HIV	2,472	Both	45–59
**Nawagi *etal***[56]	2016	2013	Katanga slum/Kampala/Uganda	women of reproductive age	STI	335	Females	18–45
**Ngaira *etal***[57]	2016	2014	Mbagathi/Nairobi/Kenya	pregnant women	HBV	287	Females	15–49
**Noubiap *et al***[58]	2013	2012	Yaoundé/Cameroon	medical students	HBV	111	Both	20–27
**Nubed *et al***[59]	2016	2014	Fako/South West Region/Cameroon	senior secondary school students	HIV	464	Both	13–25
**Ojieabu *et al***[60]	2011	2011	Sagamu/Ogun/Nigeria	Pregnant Women	HIV	403	Females	20- ≥40
**Okonkwo *et al***[61]	2017	2014	Nigeria	Traders	HBV	335	Both	median 29
**Okonkwo *et al***[62]	2018	2015	Cross River State/Nigeria	general population	HBV	1,620	Both	18- ≥18
**Oladepoand Fayemi** [63]	2011	2010	Ibadan South-West Local Government Area/Oyo/Nigeria	Secondary students	HIV	420	Both	10–19
**Omotowo *et al*** [64]	2018	2016	Enugu/Nigeria	Administration staff of a hospital	HBV	3,132	Both	18–75
**Oppong and Oti-Boadi**[65]	2013	N/A	Accra/Ghana	undergraduate private university students	HIV	324	Both	17–37
**Owusu**[66]	2015	After 2010	Cape Coast Metropolis/Ghana	primary school children	HIV	120	Both	9–13
**Oyekale**[67]	2014	2012	All regions of Cameroon	men of reproductive age	HIV	7,191	Male	15–49
**Paintsil *et al***[68]	2015	2013/2014	Accra and Ashanti/Ghana	pediatric caregivers	HIV	298	Both	32–53
**Pathmanathan *et al***[69]	2016	N/A	Malawi	General population	HIV	3,630	Both	15–49
**Poole *et al***[70]	2013	2011	Bamako/ Mali	adults and adolescents in Mali	HPV	51	Both	12–26
**Reuter *et al***[71]	2018	2013	Antsiranana/Madagascar	university students	HIV/HBV/HCV/HPV/STIs	242	Both	20–26
**Rukundo *et al***[72]	2016	2014	Kampala and Buikwe districts/Uganda	school students	HIV	245	Both	10–19
**Sahile *et al***[73]	2015	2014	Ambo/Oromia/Ethiopia	university students	HIV	1,311	Both	18–30
**Saleh *et al***[74]	2014	2011	Menoufia and Giza governorates/Egypt	residents	HCV	67	Both	18–80
**Sandqvist *et al***[75]	2011	2010	Mateete/Central Region/Uganda	pregnant women	HIV	120	Females	15–46
**Schwitters *et al***[76]	2015	2011–2013	Katutura/Namibia	young HIV-negative men	HIV	501	Male	<18
**Seyoum and Legesse**[77]	2013	2011	Harar town/Harari/Ethiopia	TB patients	HIV	415	Both	16–60
**Shiferaw *et al***[78]	2014	After 2010	Gondar/Amhara/Ethiopia	university students	HIV	384	Both	19–26
***Sultan et al***[79]	2018	2014	Cairo/Eygpt	HCV patients	HCV	203	Both	≥18
***Tarekegne et al***[80]	2018	2017	Sokota/Ethiopia	workers in an engineering Company	HIV	55	Both	≥15
**Umar and Oche**[81]	2012	2010	Sokoto/Nigeria	religious leaders	HIV	158	Male	≥30
**Wagenaar *et al***[82]	2012	2010	South Africa	Men Who Have Sex with Men	HIV	1593	Male	≥18
***Yaya et al***[83]	2018	2013	Nigeria	community dwelling women	HIV	38,948	Females	15–49
***Zungu et al***[84]	2016	2012	South Africa	medically and traditionally circumcised males	HIV	11,086	Male	≥15

### Study characteristics

The characteristics of the included studies are depicted in (Table 1), among which the oldest were published in 2010 while the most recent ones were published in 2018. Fifty one research articles determining HIV awareness level and/or knowledge and/or attitudes were included, while 14 articles determining HBV awareness level and/or knowledge and/or attitudes were included. Furthermore, 6 and 9 articles concerned of awareness level and/or knowledge and/or attitudes level regarding HCV and HPV were included, respectively. Seven articles determining STIs awareness level and/or knowledge and/or attitudes as general were also included. Two hundred questions were summarized among which 136 questions were analyzed and synthesized from included studies including the subgroup analysis. Publication bias assessment indicated no major asymmetry.

### Human immunodeficiency virus (HIV)

**Fig 2:**
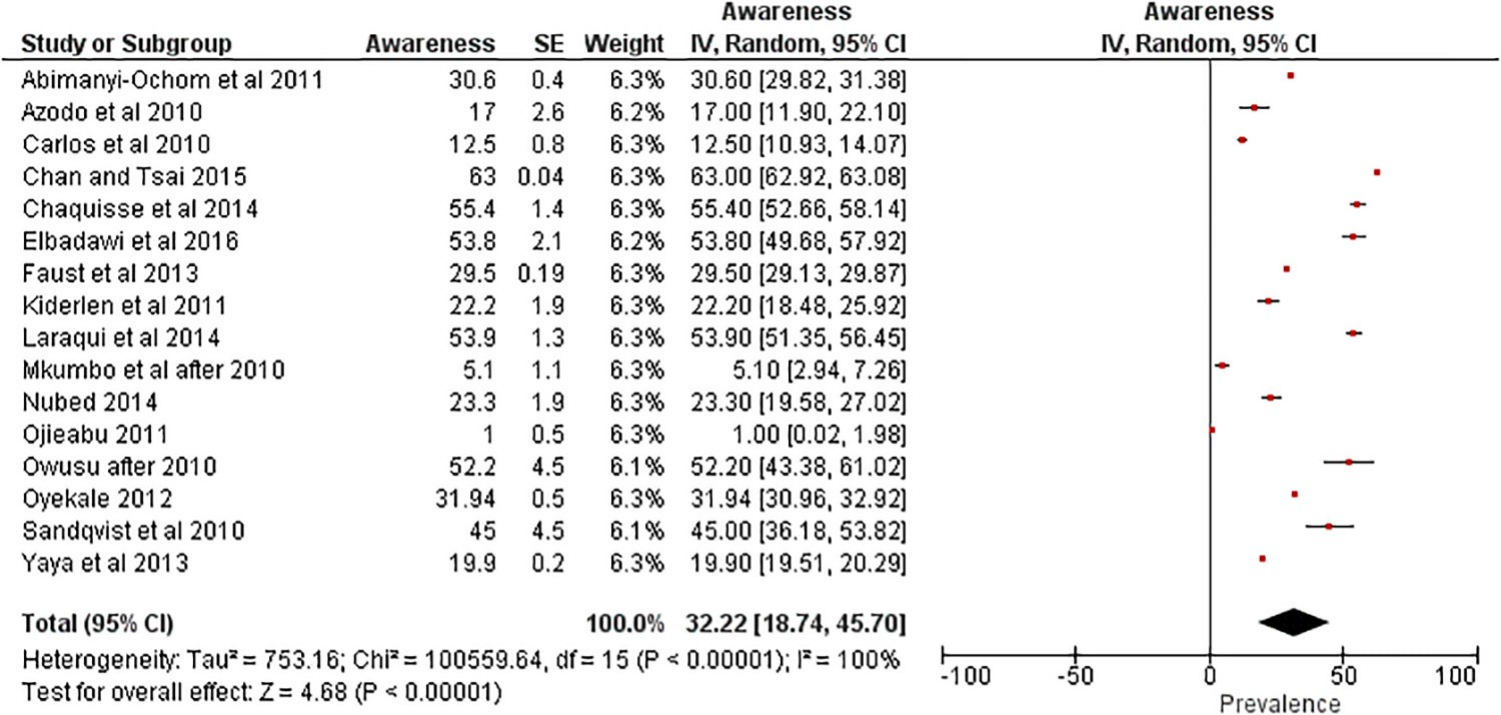
Meta analysis of 1,799,374 Africans’ yes response to the question “Does using condom prevents HIV transmission?”

Fifty oneincluded studies assessed the awareness of 1,342,002 Africans in regard to HIV in total of 35 countries, eleven studies were conducted in Nigeria [14,16–18,23,38,48,60,63,81,83], nine in South Africa [24,27,29,34,37,42,46,82,84], five in each of Ghana[19,31,65,66,68] and Ethiopia [21,73,77,78,80], four in Uganda [13,55,72,75], three in Mozambique [22,26,30], two in each of Namibia[45,76]and Cameroon [59,67], one in each of Congo [25], Sudan [33], Senegal [39], Morocco [47], Gambia [50], Tanzania [53], Madagascar [71] and Egypt [74] while a study provided awareness prevalence in 33 countries [8]. The conduction of the studies ranged from 2010 to 2017. Population under study was distributed among students and adolescents, general population, pregnant women, female sex workers, male sex workers or males who show interest of males, TB patients, seafarers and other occupations (Table 1). Majority of studies were conducted among both genders (34/51), eight studies were toward females only while nine were toward males only. Age of respondents ranged from 10 to 60 years (Table 1). Forty two questions were asked to the participants that are related to the knowledge and awareness of HIV as general, transmission routes, clinical symptoms, pathological consequences and prevention attitude, among which 31 questions were analyzed and synthesized. The question “does using condom reduces HIV transmission?” was answered by 1,316,873 Africans in Benin, Burkina Faso, Burundi, Cameroon, Chad, Comoros, Cote d’Ivoire, Democratic Republic of Congo, Ethiopia, Gabon, Ghana, Guinea, Kenya, Lesotho, Liberia, Madagascar, Malawi, Mali, Morocco, Mozambique, Namibia, Niger, Nigeria, Rwanda, São Tomé and Príncipe, Senegal, Sierra Leone, South Africa, Sudan, Swaziland, Tanzania, Togo, Uganda, Zambia and Zimbabwe; 66.8% [95% Cl; 62.6, 70.9] answered yes. The question ‘‘Is HIV contracted through Sexual intercourse?” was answered by 252,482 participants in South Africa, Ethiopia, Uganda, Madagascar, Ghana, Nigeria, Gambia, Morocco, Namibia, Senegal, Sudan and Mozambique; 72.2% [95% Cl; 64.2, 80.1] answered yes. Questions asked, their corresponding articles’ data, the pooled prevalence, the pooled prevalence after conducting sensitivity analysis andthe confidence intervals are depicted in (Table 2 & Fig 2). Heterogeneity was high in all questions (I^2^ more than 80%), except for the question “Is TB associated with HIV infection?” where I^2^ = 0%.

**Table 2:** Awareness of HIV related knowledge among Africans.

Question	Country/ies*	Study population/s	Total sample size	References	Pooled prevalence of yes response [95% Cl]	Sensitivity analysis **
**Have you heard about HIV before?**	NI, SD, CR, UG, EG, ET,	students, general population, pregnant Women, men of reproductive age, newly married couples without formal education, TB patients, workers	65,562	[16,33,38,60,67,72,74,77,80]	92.2 [89.6, 94.8]	94.4 [91.8, 97.0]
**Can a healthy person be HIV infected?**	SD, BE, BF, BR, CR, CH, Comoros, DC, IC, DC, ET, GB, GH, GU, KY, LE, LI MD, MW, ML, MZ, NM, NG, NI, RW, RS, SN, SL, SW, TA, TO, UG, ZM, ZI,	community dwelling women, MSM, pediatric caregivers, men of reproductive age, Students, employees in truck transport sector, pregnant women, Outpatients and blood donors, General population	1,306,715	[8,13,67,68,72,82,83,19,25,30,33,38,45,53,59]	69.6 [66.6, 72.7]	70.9 [67.9, 74.0]
**Do you perceive risk of contracting HIV?**	SA, NM, GH, NI.	students, men who indicated an interest in men, adolescents, general population, medically and traditionally circumcised males	13,073	[23,27,31,76,84]	39.2 [-5.3, 83.8]	36.9 [13.9, 60.0]
**Is it possible to cure HIV?**	NI, MZ, SA, TA, CR, UG	Students, Traditional healers, pregnant women, General population	813,382	[16,22,30,42,53,59,72]	41.9 [18.7, 65.1]	39.9 [22.4, 57.4]
**Do you know a place to get HIV testing?**	NI, GH	students, general population	56,631	[38,65]	85.7 [59.7, 111.7]	
**Is social stigma a barrier to HIV testing?**	SA	General population	1,146	[29]	37.4 [34.6, 40.1]	
**Is ignorance causes stigma to HIV testing?**	SA	General population	1,146	[29]	46.3 [43.5, 49.0]	
**Is HIV transmission possible through mosquito bites?**	SD, BE, BF, BR, CR, CH, Comoros, DC, IC, DC, ET, GB, GH, GU, KY, LE, LI MD, MW, ML, MZ, NM, NG, NI, RW, RS, SN, SL, SW, TA, TO, UG, ZM, ZI,	community dwelling women, pregnant women, men of reproductive age, Students, seafarers, employees in truck transport sector, Outpatients and blood donors, General population	1,307,443	[8,13,23,25,26,33,38,45,47,53,59,60,66,67,75,83]	32.2 [18.7, 45.7]	32.8 [18.9, 46.6]
**Is HIV transmission possible through sharing food?**	SD, BE, BF, BR, CR, CH, Comoros, DC, IC, DC, ET, GB, GH, GU, KY, LE, LI MD, MW, ML, MZ, NM, NG, NI, RW, RS, SN, SL, SW, TA, TO, UG, ZM, ZI,	community dwelling women, men of reproductive age, Students, employees in truck transport sector, seafarers, general population	1,302,877	[8,13,33,38,39,45,53,59,66,67,83]	27.3 [2.6, 51.9]	26.49 [-0.7, 53.7]
**Is HIV transmission possible during pregnancy?**	UG, NI, ET, MZ, SA, SN, NM, MR, TA, GH, EG,	community dwelling women, MSM, workers, pregnant women, newly married couples without formal education, pediatric caregivers, undergraduate university students, General population, Students, pregnant women, clients presenting for Male medical circumcision, employees in truck transport sector, seafarers,	115,604	[13,14,16,21,26,37–39,45,47,53,60,65,68,74,75,80,82,83]	57.0 [52.4, 61.7]	57.6 [53.1, 62.0]
**Is HIV transmission possible during delivery?**	UG, NI, MZ, TA, EG,	community dwelling women, newly married couples without formal education, prenatal care in women, Students, General population	107,684	[13,14,30,38,53,74,83]	66.6 [50.9, 82.3]	62.9 [45.9, 79.8]
**Is HIV transmission possible during breastfeeding?**	UG, NI, GH, MZ, NM, MR, TA, EG,	community dwelling women, newly married couples without formal education, seafarers, employees in truck transport sector, Pregnant women, Students, General population	111,180	[13,14,19,26,30,38,45,47,53,74,75,83]	73.4 [65.4, 81.3]	75.2 [66.9, 83.5]
**Is HIV contracted through Sexual intercourse?**	SA, ET, UG, MD, GH, NI, GA, MR, NM, SN, SD, MZ,	Students,Adolescents, Pregnant women, workers, MSM, pediatric caregivers, prenatal care in women, FSW, clients presenting for Male medical circumcision, seafarers, employees in truck transport sector,	252,482	[14,16–19,23,26,30,33,37,39,45,47,48,50,60,65,66,68,71,72,75,80,82]	72.2 [64.2, 80.1]	73.3 [65.3, 81.3]
**Is HIV contracted through sharing sharp unsterilized objects?**	MZ, SN, MR, NI, GH	Students, Pregnant women, prenatal care in women, seafarers, FSW, Pregnant Women, primary school children	4,268	[16,26,30,39,47,48,60,66]	50.8 [22.1, 79.4]	51.5 [23.9, 79.1]
**Is HIV contracted through transfusion with unscreened blood?**	NI, GH, SD, SA, UG, ET	Students, clients presenting for Male medical, FSW, Pregnant Women, workers	2,595	[16,19,23,33,37,48,60,66,72,80]	54.3 [28.1, 80.6]	55.6 [28.0, 83.2]
**Is HIV contracted through shaking hands?**	NI, MZ, NM, TA, UG	Students, Pregnant women, employees in truck transport sector	2,937	[16,23,26,30,45,53,75]	18.2 [7.6, 28.9]	19.3 [8.6, 30.0]
**Is HIV contracted through witchcraft and other spiritual factors?**	GH, DC, MZ, NM, TA, NI	Students, Outpatients and blood donors, prenatal care in women, general population, employees in truck transport sector, community dwelling women	98,442	[19,25,30,38,45,53,66,83]	36.1 [29.1, 43.0]	34.3 [25.3, 43.4]
**Is HIV contracted through Intravenous needles?**	NI, MZ, SD, SA, GA, TA, GH, UG, ET	students, Pregnant women, clients presenting for Male medical circumcision, FSW, MSM, workers in China first high way engineering Company	4,522	[23,26,72,75,80,30,33,37,48,50,53,60,65]	64.7 [45.4, 84.1]	67.6 [52.1, 83.0]
**Is HIV transmission possible during blood donation?**	NI, MZ	Pregnant women, Dental tech students	1,384	[23,26]	73.8 [65.9, 81.7]	
**Is HIV transmission possible through sharing of cups/plates?**	NI, MZ, GH, UG, SA	Students, Pregnant women, pediatric caregivers, MSM	302,165	[14,23,26,30,66,68,75,82]	20.9 [9.7, 32.2]	19.2 [7.4, 31.0]
**Is HIV transmission possible through hugging and kissing?**	NI, DC, MZ, SA, TA, GH, UG	Students, Outpatients and blood donors, clients presenting for Male medical circumcision, pediatric caregivers, pregnant women, MSM	6,287	[14,25,26,37,53,66,68,75,82]	26.0 [15.1, 37.0]	25.8 [16.4, 35.1]
**Can coughing and sneezing spread HIV?**	MZ, GH, SA	MSM, pediatric caregivers, pregnant women	2,239	[30,68,82]	14.9 [6.9, 22.9]	
**Is HIV transmission possible through toilets?**	NI, MZ	Pregnant women, Students	1,786	[14,26]	43.7 [34.9, 52.6]	
**Is HIV transmission possible through tattoos or perforation?**	MZ	Pregnant women	1,186	[26]	70.0 [67.6, 72.3]	
**Is oral candidiasis associated with HIV infection?**	SA	General population	2,477	[24]	14.4 [13.0, 15.7]	
**Is herpes zosters associated with HIV infection?**	SA	General population	2,477	[24]	17.0 [15.6, 18.3]	
**Is TB associated with HIV infection?**	SA	General population, home-based carers	2,621	[24,34]	18.0 [16.6, 19.3]	
**Is wasting associated with HIV infection?**	SA	General population	2,477	[24]	23.8 [22.2, 25.3]	
**Are sores or abscesses associated with HIV infection?**	SA	General population	2,477	[24]	23.0 [21.4, 24.5]	
**Is acute respiratory tract infection associated with HIV infection?**	SA	General population	2,477	[24]	26.7 [27.9, 31.4]	
**Is constant diarrhea associated with HIV infection?**	SA	General population	2,477	[24]	17.7 [16.3, 19.0]	
**Do you consider loss of body weight a sign of AIDS?**	SA	General population	2,477	[24]	43.7 [39.9, 47.4]	
**Does using condom reduces HIV transmission?**	BE, BF, BR, CR, CH, Comoros, DC, IC, DC, ET, GB, GH, GU, KY, LE, LI MD, MW, ML, MZ, NM, NG, NI, RW, RS, SN, SL, SW, TA, TO, UG, ZM, ZI, SD, MR,	General population, Students, Outpatients and blood donors, adolescents, seafarers, rural based traditional healers, employees in truck transport sector, STI service attendees, FSW, Pregnant Women, men of reproductive age, pediatric caregivers, workers, religious leaders, MSM, community dwelling women.	1,316,873	[8,13,16,19,23,25,29–31,33,38,39,42,45–48,53,59,60,65–69,75,80–83]	66.8 [62.6, 70.9]	68.0 [63.9, 72.1]
**Is having one sexual partner prevent HIV transmission?**	BE, BF, BR, CR, CH, Comoros, DC, IC, DC, ET, GB, GH, GU, KY, LE, LI, MD, MW, ML, MZ, NM, NG, NI, RW, RS, SN, SL, SW, TA, TO, UG, ZM, ZI, SD, MR,	General population,Students, Outpatients and blood donors, adolescents, seafarers, rural based traditional healers, employees in truck transport sector, STI service attendees, FSW, Pregnant Women, men of reproductive age, pediatric caregivers, workers, religious leaders, MSM, community dwelling women	1,316,873	[8,13,16,19,23,25,29–31,33,38,39,42,45–48,53,59,60,65–69,75,80–83]	67.6 [64.7, 70.4]	70.1 [67.7, 72.6]
**Is abstinence the best way of preventing HIV?**	GH, NI, SN, SA, TA, CR.	Students, seafarers, rural based traditional healers, religious leaders	3,794	[19,23,31,39,42,53,59,63,65,66,81]	64.5 [48.6, 80.4]	
**Do you consider showering or washing one’s genitals / private parts after sex keeps a person from getting HIV?**	GH, MZ, SA.	Pregnant women, pediatric caregivers, MSM.	2,239	[30,68,82]	19.7 [0.2, 39.3]	
**Did you practice HIV testing during last pregnancy?**	ET	Pregnant women	1,325	[21]	89.3 [87.5, 91.0]	
**Is it ok for a person with HIV to teach?**	NI, GH, UG	General population, primary school children, community dwelling women	50,037	[13,66,83]	44.8 [10.1, 79.4]	
**Is it ok to care for a relative with HIV in household?**	UG, NI	community dwelling women, General population	49,917	[13,83]	62.9 [11.1, 114.7]	
**Is it ok to buy vegetables from a vendor with HIV?**	UG, NI	community dwelling women, General population	49,917	[13,83]	57.1 [25.7, 88.5]	
**Do you have feelings of high stigma towards HIV-infected patients?**	UG	General population, older adults	2,472	[55]	31.1 [29.3, 32.8]	
**Did you used Condom in the last 12 months?**	ET	university students,	1,695	[73,78]	51.6 [25.2, 77.9]	
**Is HIV transition possible through oral six?**	SA, MZ	Men Who Have Sex with Men, Pregnant women	4,227	[26,30,82]	34.1 [-0.4, 68.7]	

* Country codes are as follow:

Benin = BE, Burkina Faso = BF, Burundi = BR, Cameroon = CR, Democratic Republic of the Congo = DC, Egypt = EG, Ethiopia = ET, Gabon = GB, Gambia = GA, Ghana = GH, Guinea = GU, Kenya = KY, Lesotho = LE, Liberia = LI, Madagascar = MD, Malawi = MW, Mali = ML, Morocco = MR, Mozambique = MZ, Namibia = NM, Niger = NG, Nigeria = NI, Chad = CH, Republic of Sao Tome and Principe = RS, Rwanda = RW, Senegal = SN, Sierra Leone = SL, South Africa = SA, Sudan = SD, Swaziland = SW, Tanzania = TA, Togo = TO, Uganda = UG, Zambia = ZM, Zimbabwe = ZI

** Sensitivity analysis was conducted wherever population of proposed high level of knowledge or population proposed to behave in indifference was participated in a question.

#### Nigeria

Eleven included studies in regard to HIV were conducted among Nigerians representing a total population of 98,279 participants; three studies were conducted in Oyo State [14,16,63] and one in each of Osun State [17], Ekiti State [18], Enugu State [23], Kano State [48], Sokoto State[81]and Ogun State [60]. Two studies were nationally representative and participants were from different States [38,83]. The oldest among the study included were conducted in 2010 while the newest were conducted in 2013 (S3 Table). Population under study was found to be mainly students and adolescents (6/11), while one was toward each of pregnant women, religious leaders, general population, female sex workers and community dwelling women (S3 Table). Majority of studies were conducted among both genders (7/11), three studies were toward females only while one study included only males. Age of respondents ranged from 12 to 49 years. Twenty two questions were asked to the participants that are related to the knowledge and awareness of HIV as general, transmission routes, clinical symptoms, pathological consequences and prevention attitude, among which 18 questions were analyzed and synthesized. The question ‘‘ Does using condom reduces HIV transmission?” was answered by 57,430 participants; 52,6% [95% Cl; 44.4, 60.9] answered yes. The question ‘Is HIV can be transmitted through mosquito bites?” was answered by 95,856 participants; 16.8% [95% Cl; 6.7, 26.9] answered yes. Questions asked, their corresponding study’s characteristics, the pooled prevalence and the confidence intervals are depicted in (Table 3). Heterogeneity was high in all questions (I^2^ more than 80%).

**Table 3:** Awareness of HIV related knowledge among Nigerian population.

Questions	Study population/s	Total sample size	References	Pooled prevalence of yes response [95% Cl]
**Does using condom reduces HIV transmission?**	Students, general population, FSW, Pregnant Women, religious leaders, community dwelling women	96,378	[16,23,38,48,60,81,83]	52.6 [44.4, 60.9]
**Is having one sexual partner will reduce HIV transmission?**	Students, general population, Pregnant Women, community dwelling women	96,096	[16,23,38,60,83]	57.6 [48.5, 66.8]
**Does a Healthy person can be HIV infected?**	general population, community dwelling women	95,255	[38,83]	76.9 [68.4, 85.4]
**Can HIV be transmitted through mosquito bites?**	Dental tech students, general population, Pregnant Women, community dwelling women	95,856	[23,38,60,83]	16.8 [6.7, 26.9]
**Can HIV be transmitted through sharing food?**	general population, community dwelling women	95,255	[38,83]	16.3 [11.6, 21.0]
**Is it OK for a person with HIV to teach?**	community dwelling women	38,948	[83]	51.6%
**Is it OK to care for a relative with HIV in household?**	community dwelling women	38,948	[83]	63.0%
**Is it ok to buy vegetables from a vendor with HIV?**	community dwelling women	38,948	[83]	41.0%
**Is HIV transmission possible during pregnancy?**	Students, general population(NDHS), Pregnant Women, community dwelling women	96,498	[14,16,38,60,83]	45.9 [40.0, 50.8]
**Is HIV transmission possible during delivery?**	Students, general population, community dwelling women	95,855	[14,38,83]	56.6 [54.3, 59.9]
**Is HIV transmission possible during breastfeeding?**	Students, general population, community dwelling women	95,855	[14,38,83]	66.2 [62.9, 70.6]
**Have you heard about HIV before?**	Students, general population, Pregnant Women	56,950	[16,38,60]	94.9 [91.8, 97.0]
**Is HIV contracted through Sexual intercourse?**	Students, Adolescents, FSW, Pregnant Women,	2,446	[14,16–18,23,48,60]	68.9 [41.0, 94.8]
**Is HIV contracted through Sharing sharp unsterilized objects?**	Students, FSW, Pregnant Women	767	[16,48,60]	46.7 [10.9, 83.5]
**Is HIV contracted through unscreened blood?**	Students, FSW, Pregnant Women	965	[16,23,48,60]	51.7 [4.2, 97.2]
**Is HIV contracted through shaking hands?**	Students, Dental tech students	438	[16,23]	2.9 [-2.0, 7.8]
**Is HIV contracted through witchcraft and other spiritual factors?**	general population(NDHS),community dwelling women	95,255	[38,83]	24.5 [11.1, 36.9]
**Is abstinence the best way of preventing HIV?**	students, religious leaders	776	[23,63,81]	55.7 [14.2, 97.2]
**Is HIV contracted through intravenous needles?**	Dental tech students, FSW, Pregnant Women	725	[23,48,60]	61.7 [7.9, 115.6]
**Is HIV contracted through sharing of cups/plates?**	Students	798	[14,23]	32.0 [-3.2, 68.1]
**DO you knows a place to get HIV testing?**	general population	56,307	[38]	68.9

#### South Africa

Nine included studies in regard to HIV were conducted among South Africans representing a total population of 17,320 participants; two studies were conducted in KwaZulu-Natal province[24,37] and one was conducted in each of Gauteng Province [29], Northern Cape province [42], North West province [34], two studies were toward online internet users [27,82], one study was conducted in Eastern Cape, Western Cape, Free State and Gauteng Provinces [46]while another study was nationally representative [84] (S4 Table). The oldest among the study included was conducted in 2010 while the newest was conducted after 2010. Population under study was distributed among circumcised males, men who have sex with men or indicated interest in men, general population and home-based carers (S3 Table). Majority of studies were conducted among both genders (5/9), while four were toward males only (S4 Table). Age of respondents was from 15 to more than 25 years. Thirty two questions were asked to the participants that are related to the knowledge and awareness of HIV as general, transmission routes, clinical symptoms and prevention attitude, among which 16 questions were analyzed and synthesized. The question ‘Does using condom reduces HIV transmission?” was answered by 3,979 participants; 64.6% [95% Cl; 31.0, 97.1] answered yes. The question ‘‘Do you perceive risk of contracting HIV?” was answered by 11,472 participants; 42.5% [95% Cl; -36.4, 120.5] answered yes. Questions asked, their corresponding study’s characteristics data, the pooled prevalence and the confidence intervals are depicted in (Table 4). Heterogeneity was high in all questions (I^2^ more than 80%).

**Table 4:** Awareness of HIV related knowledge among South African population.

Question	Study population/s	Total sample size	References	Pooled prevalence of yes response [95% Cl]
**Does Using condom reduces HIV transmission?**	General population, home-based carers, rural based traditional healers (THs), STI service attendees, Men Who Have Sex with Men (MSM).	3,979	[29,42,46,82]	64.6 [31.0, 97.1]
**Does having one sexual partner reduces HIV transmission?**	rural based traditional healers, Men Who Have Sex with Men (MSM).	1,779	[42,82]	83.5 [55.3, 111.7]
**Can a healthy person can be HIV infected?**	MSM	1,593	[82]	94,0% [88.1, 98.3]
**Is HIV transmission possible during pregnancy?**	General population, MSM	1,987	[37,82]	77.3 [53.2, 101.4]
**Is HIV contracted through Sexual intercourse?**	General population, MSM	1,987	[37,82]	91.5 [80.8, 102.2]
**Is HIV contracted through sharing cups/plates?**	(MSM	1,593	[82]	7 [6.3, 8.7]
**Do you perceive risk of contracting HIV?**	men who indicated an interest in men, medically and traditionally circumcised males	11,472	[27,84]	42.5 [-36.4, 120.5]
**Is ignorance the reason that stigma and discrimination related to HIV testing?**	General population	1,146	[29]	46,4 [39.1, 52.1]
**Are the social stigma and discrimination barriers to HIV testing?**	General population	1,146	[29]	37,3 [35.1, 39.4]
**Is your perception of the risk of getting HIV infection low?**	General population	1,146	[29]	51,3 [50.7, 53.0]
**Is HIV transmission possible through hugging and kissing**	General population, MSM	1,987	[37,82]	4.4 [-0.3, 10.1]
**Do you consider loss of body weight a sign of AIDS?**	General population, home-based caregivers.	2,477	[24]	34.4[29.5, 41.8]
**Is HIV transmission possible through oral six?**	MSM	1,593	[82]	18.0 [15.8, 25.6]
**Is HIV transmission possible through coughing and sneezing**	MSM	1,593	[82]	8,0 [2.1, 15.5]
**Do you consider pulling the penis out before a man climaxes/cums keeps his partner from getting HIV during sex?**	MSM	1,593	[82]	4.0 [3.2, 6.5]
**Does showering or washing one’s genitals / private parts after sex keeps a person from getting HIV**	MSM	1,593	[82]	1.0 [-0.5, 3.9]
**Do you consider Oral candidiasis a sign of AIDS?**	General population	2,477	[24]	14.0 [12.5, 18.6]
**Do you consider Herpes zoster a sign of AIDS?**	General population	2,477	[24]	17.0 [16.8, 18.7]
**Do you consider TB a sign of AIDS?**	General population, home-based carers	2,621	[24,34]	18.0 [16.5, 19.5]
**Do you consider Wasting a sign of AIDS?**	General population	2,477	[24]	23.0[20.5, 14.0]
**Do you consider Sores/abscesses a sign of AIDS?**	General population	2,477	[24]	23 [21.5, 25.6]
**Do you consider acute respiratory tract infection a sign of AIDS?**	General population	2,477	[24]	29.0 [25.3, 32.6]
**Do you consider constant diarrhea abscesses a sign of AIDS?**	General population	2,477	[24]	17.0 [14.9, 20.9]

#### Adolescents and young people

The study participants’ age were greater than 14and less than 25 years in thirteen HIV-related included studies, representing a total population of 5,908 participants; five studies were conducted in Nigeria [14,16–18,63], three in Ghana [19,31,66], two in Mozambique [26,30], and one in each of Cameroon [59], Madagascar [71]and Uganda [72]. Majority of studies were toward students and adolescents (11/13) while two studies were conducted among pregnant women. Majority of studies were conducted among both genders (11/13), while two were toward females only (pregnant women) (S5 Table). Age of respondents was from 12 to 25 years. Twenty two questions were asked to the participants that are related to the knowledge and awareness of HIV as general, transmission routes, clinical symptoms and prevention attitude, among which 21 questions were analyzed and synthesized. The question ‘‘Do you think HIV is contracted through Sexual intercourse?” was answered by 4,122 participants; 67.1% [95% Cl;50.6, 84.6] answered yes. The question ‘‘Do you think sharing cups/plates can transmit HIV?” was answered by 2,254 participants; 33.1% [95% Cl; 9.3, 57.9] answered yes. Questions asked, their corresponding studies’ characteristics, the pooled prevalence and the confidence intervals are depicted in (Table 5). Heterogeneity was high in all questions (I^2^ more than 80%).

**Table 5:** Awareness of HIV related knowledge among adolescents in Africa.

Question	Country/ies	Study population	Total sample size	First author’s last name	Pooled prevalence of yes response [95% Cl]
**Does using condom reduces HIV transmission?**	Nigeria, Ghana, Mozambique, Cameroon	Students, prenatal care in women, adolescents, general population	2,214	[16,19,30,31,59]	75.5 [41.2, 109.9]
**Does having one sexual partner reduces HIV transmission?**	Nigeria, Mozambique, Cameroon, Ghana	Students, prenatal care in women	1,172	[16,30,59,66]	48.5 [2.1, 94.9]
**Can a Healthy person be HIV infected?**	Ghana, Mozambique, Cameroon, Uganda	Students, prenatal care in women	1,317	[19,30,59,72]	60.5 [23.8, 97.2]
**Is HIV transmission possible through mosquito bites?**	Mozambique, Cameroon,Ghana	Pregnant women, students	1,770	[26,59,66]	43.4 [19.0, 67.9]
**Is HIV transmission possible during pregnancy?**	Nigeria, Mozambique	Students, Pregnant women	2,026	[14,16,26]	58.0 [29.2, 86.9]
**Is HIV transmission possible through sharing food?**	Cameroon, Ghana	students	584	[59,66]	27.1 [-11.1, 65.3]
**Is HIV transmission possible through Delivery?**	Nigeria, Mozambique	Students, pregnant women	948	[14,30]	55.6 [52.4, 59.7]
**Is HIV transmission possible through Breastfeeding?**	Nigeria, Ghana, Mozambique, Mozambique	Students, pregnant women	2,394	[14,19,26,30]	74.5 [57.1, 91.0]
**Have You heard about HIV before?**	Nigeria, Uganda	Students	485	[16,72]	93.5 [89.5, 97.5]
**Is it possible to cure HIV?**	Nigeria, Mozambique, Cameroon, Uganda	Students, prenatal care in women	1,297	[16,30,59,72]	46.9 [13.2, 80.5]
**Is HIV contracted through Sexual intercourse?**	Nigeria, Ghana, Mozambique, Madagascar, Uganda	Students, Adolescents, Pregnant women	4,122	[14,16–19,26,30,66,71,72]	67.1 [50.6, 84.6]
**Is HIV contracted through transfusion with unscreened blood?**	Nigeria, Ghana, Uganda	Students	865	[16,19,66,72]	30.9 [-3.9, 65.7]
**Is HIV contracted through Sharing sharp unsterilized objects?**	Nigeria, Mozambique, Ghana	Students, Pregnant women	1,894	[16,26,30,66]	54.7 [29.6, 78.8]
**Is HIV contracted through shaking hands?**	Nigeria, Mozambique	Students, Pregnant women	1,774	[16,26,30]	19.3 [-0.1, 39.8]
**Is HIV contracted through witchcraft and other spiritual factors?**	Ghana, Mozambique	Students, pregnant women	728	[19,30,66]	42.6 [15.5, 69.7]
**Is Abstinence the best way of preventing HIV/AIDS?**	Ghana, Cameroon, Nigerian city, Nigeria	Students, adolescents, general population	2,166	[19,31,59,63,66]	56.1 [26.6, 85.6]
**Is HIV transmission possible through intravenous needlesticks?**	Mozambique, Mozambique, Uganda	Pregnant women, school students	1,779	[26,30,72]	51.6 [25.2, 77.9]
**Is HIV transmission possible through sharing cups/plates?**	Nigeria, Mozambique, Ghana	Students, Pregnant women	2,254	[14,26,30,66]	33.5 [9.4, 57.5]
**Is HIV transmission possible through hugging and kissing?**	Nigeria, Mozambique, Ghana	Students, Pregnant women	1,906	[14,26,66]	25.8 [-4.7, 56.3]
**Is HIV transmission possible through oral six?**	Mozambique	Pregnant women	1,534	[26,30]	42.2 [-9.1, 93.5]
**Is HIV transmission possible through sharing toilets?**	Nigeria, Mozambique	Students, Pregnant women	1,786	[14,26]	43.7 [34.9, 52.6]
**Is HIV transmission possible through tattoos or perforations?**	Mozambique	Pregnant women	1,186	[26]	74.0 [71.6, 76.3

#### Awareness of HIV related to demographic characteristics

Media (as general) was the main source of information of participants reported in several studies [17,47,74]. However, other studies among students reported that school is the main source of information not media [22,75]. Health professionals was the least mentioned source of information in the study of Saleh and colleagues [74].

Chaquisse and colleagues in their recently published study (2018) determined women’s age as not significantly associated with HIV and HBV knowledge. Moreover, they determined thatto have heard about HIV/AIDS, Syphilis, Gonorrhoea, Hepatitis B or Hepatitis C, was associated with better knowledge about HIV transmission modes [26].

Two studies indicated a statistically significant difference in the HIV/AIDS knowledge scores and the marital/ relationship status [38,65]. Nevertheless, another study indicated that no relation exists [55]. This last study also reported that stigma toward HIV was significantly associated with knowledge scores of HIV, education level and sex, while place of residence (rural versus urban) is not [55].

One study concluded that Comprehensive knowledge of HIV is significantly associated with more media items and fewer children at home [30].

Regarding religion, Christians compared to Muslims have been found to significantly have better knowledge of HIV/AIDS. Nevertheless, another study found that Muslim students scored higher on HIV/AIDS knowledge than Christian students[65,81].

Several studies indicated that the level of education and age have a significant association with the knowledge of HIV transmission [21,39,48]. Additionally, one study [81] agreed that only education level is associated, while another agreed that only age is associated [77]. Nevertheless, Faye and colleagues only concluded that marital status is associated to the knowledge of HIV transmission [39].

Seyoum and colleagues concluded that female participants who heard about HIV was significantly higher than that of the male participants. Moreover, there was a significant difference between males and females who suggested unsafe sexual intercourse as mode of transmission of HIV[77]. However, Yaya and colleagues found that the majority (82.5%) of participants (females) (N = 32,123) believe on contracting the virus via supernatural means [83].

### Hepatitis B virus (HBV)

Fourteen included studies assessed the awareness of 9,446 Africans in regard to HBV, three studies were conducted in each of Nigeria [61,62,64], Cameroon [20,40,58] and Ghana [12,15,28], two in Ethiopia [32,52], one in each of Kenya, Mozambique and Madagascar [26,57,71]. The oldest among the study included was conducted in 2010 while the newest was conducted in 2016 (Table 1). Population under study was found to be mainly students and adolescents and pregnant women (10/14), one study was targeting each of non medical staff of health facilities, general population, barbers and traders (Table 1). Majority of studies were conducted among both genders (8/14), five studies were toward females only (pregnant women) while one study included only males (barbers). Age of respondents ranged from 10 to 75 years. Fifteen questions were asked to the participants that are related to the knowledge and awareness of HBV as general, transmission routes, clinical symptoms, pathological consequences and prevention attitude, among which 13 questions were analyzed and synthesized. The question ‘‘Do you know HBV?” was answered by 4,066 participants in Ghana, Mozambique, Ethiopia, Nigeria and Madagascar; 53.8% [95% Cl; 27.6, 79.9] answered yes. The question ‘‘Does sexual contact is a possible route of HBV transmission?” was answered by 7,490 participants in Ghana, Mozambique, Ethiopia, Cameroon and Nigeria; 42.5% [95% Cl; 20.4, 64.7] answered yes. Questions asked, their corresponding articles’ data, the pooled prevalence and the confidence intervals are depicted in (Table 6). Heterogeneity was high in all questions (I^2^ more than 80%).

**Table 6:** Awareness of HBV related knowledge among Africans.

Question	Country/ies	Study population/s	Total sample size	References	Pooled prevalence of yes response [95% Cl]
**Do you know HBV?**	Ghana, Mozambique, Ethiopia, Kenya, Nigeria, Madagascar	Barbers, Pregnant women, students, general population	4,570	[12,15,26,28,52,57,62,71]	53.8 [27.6, 79.9]
**Can HBV damage liver?**	Ghana, Ethiopia, Cameroon, Nigeria	students, pregnant women, general population	2,735	[28,32,40,52,62]	61.4 [31.3, 91.5]
**Is Blood transfusion a possible route of HBV transmission?**	Ghana, Mozambique, Ethiopia, Cameroon, Nigeria	Barbers, Pregnant women, Students, traders, general population, Administration staff of a hospital	7,490	[15,26,32,40,52,58,61,62,64]	56.1 [28.6, 83.6]
**Is reusing needles a possible route of HBV transmission?**	Ghana, Mozambique, Ethiopia, Cameroon, Nigeria	Barbers, Pregnant women, Students, general population, Administration staff of a hospital	7,155	[15,26,32,40,52,58,62,64]	52.7 [26.9, 78.4]
**Is sharing blades a possible route of HBV transmission?**	Ghana, Mozambique, Ethiopia, Cameroon, Nigeria	Barbers, Pregnant women, medicine and health science students, traders, Administration staff of a hospital	5,437	[15,26,32,40,61,64]	39.7 [4.4, 75.1]
**Is tattooing a possible route of HBV transmission?**	Ghana, Mozambique, Nigeria	Barbers, Pregnant women, traders, general population	3,341	[15,26,61,62]	28.2 [5.0, 51.3]
**Is sexual contact a possible route of HBV transmission?**	Ghana, Mozambique, Ethiopia, Cameroon, Nigeria	Barbers, Pregnant women, Students, traders, general population, Administration staff of a hospital	7,490	[15,26,32,40,52,58,61,62,64]	42.5 [20.4, 64.7]
**Are mosquito bites possible route of HBV transmission?**	Mozambique, Nigeria	Pregnant women, general population	2,806	[26,62]	28.4 [-3.8, 60.8]
**Is mother to child a possible route of HBV transmission?**	Mozambique, Ethiopia, Nigeria	pregnant women, Students, general population	3,745	[26,28,32,52,62]	57.5 [35.9, 79.1]
**Can Hepatitis B transmit through feco-oral route?**	Ethiopia, Cameroon	medical students	519	[32,58]	48.6 [6.5, 90.7]
**Is toothbrush sharing a possible route of HBV transmission?**	Nigeria	General population	1,620	[62]	49.0 [46.6, 51.3]
**Can HBV be asymptomatic?**	Ethiopia, Cameroon	pregnant women, medicine and health science students	793	[20,28,32,40]	58.1 [23.3, 92.8]
**Can HBV be prevented by avoiding casual sex or multi sexual partnership?**	Ethiopia	medicine and health science students	408	[32]	87.0 [83.8, 90.1]
**Do you know HBV vaccination?**	Ethiopia, Cameroon	pregnant women, Students	1,226	[28,32,40,52,58]	72.3 [50.6, 94.0]

#### Awareness of HBV related to demographic characteristics

Abdulai and colleagues in their study among pregnant women determined that level of education and occupation are significantly associated to hepatitis B awareness[12]. Frambo and colleagues among the same population concluded that education is significantly associated to the level of awareness as well[40]. Furthermore, Ngaira and colleagues assessed the awareness as well as vaccination status among the same population (pregnant women) and indicated a significant difference between vaccine uptake and education[57].

Noubiap and colleagues assessed HBV vaccine uptake but among medical students, and indicated that duration of study but not age or vaccination status are significantly correlated. Nevertheless Okonkwo and colleagues in their study among traders concluded that knowledge of the nature of HBV virus varied significantly according to age[58,61].

### Hepatitis C virus (HCV)

Sixincluded studies assessed the awareness of 2,306 Africans in regard to HCV, two studies were conducted in Egypt [74,79] and one in each of Ghana [15], Mozambique [26], Ethiopia [32] and Madagascar [71]. The oldest among the study included was conducted after 2010while the newest was conducted in 2015 (Table 1). Population under study was distributed among students and adolescents, general population, HCV positive patients, pregnant women and barbers (Table 1). Four studies were conducted among both genders, one toward females only and one toward males only (Table 1). Age of respondents range from 18 to 80 years. Seventeen questions were asked to the participants that are related to the knowledge and awareness of HCV as general, transmission routes, clinical symptoms, pathological consequences and prevention attitude, among which 10 questions were analyzed and synthesized. The question ‘‘Is sexual contact a possible route of HCV transmission?” was answered by 1,997 Africans in Ghana, Mozambique, Ethiopia and Egypt; 30.6% [95% Cl; 2.0, 59.1] answered yes. The question ‘‘Would Hepatitis C infection be prevented by vaccination?” was answered by 611 participants in Ethiopia and Egypt; 42.0 [95% Cl; 8.7, 75.3] answered yes. Questions asked, their corresponding articles’ data, the pooled prevalence and the confidence intervals are depicted in (Table 7). Heterogeneity was high in all questions (I^2^ more than 80%).

**Table 7:** Awareness of HCV related knowledge among Africans.

Question	Country/ies	Study population/s	Total sample size	References	Pooled prevalence of yes response [95% Cl]
Have you ever heard about viral hepatitis C?	Ghana, Mozambique, Egypt	Barbers, Pregnant women, university students, HCV patients	1,831	[15,26,71]	20.2 [5.4, 35.0]
Do you know what causes HCV infection?	Egypt	General population	67	[74]	87.0 [68.2, 87.7]
Can HCV be cured?	Egypt	General population	67	[74]	39.0 [27.4, 50.5]
Can HCV causes liver cancer?	Ethiopia, Egypt	medicine and health science students, HCV patients	611	[32,79]	89.5 [87.1, 91.8]
Is blood transfusion a possible route of HCV transmission?	Ghana	Barbers	200	[15,79]	86.4 [81.7, 91.1]
Is reusing needles a possible route of HCV transmission?	Ghana, Mozambique, Ethiopia, Egypt	Barbers, Pregnant women, medicine and health science students, HCV patients	1,997	[15,26,32,79]	68.8 [16.9, 120.6]
Is sharing blades a possible route of HCV transmission?	Ghana, Mozambique, Egypt	Barbers, Pregnant women, HCV patients	1,589	[15,26,79]	54.7 [-9.4, 118.9]
Is tatooinga possible route of HCV transmission?	Ghana, Mozambique	Barbers, Pregnant women	1,386	[15,26]	21.6 [19.4, 23.7]
Is sexual contact a possible route of HCV transmission?	Ghana, Mozambique, Ethiopia, Egypt	Barbers, Pregnant women, medicine and health science students, HCV patients	1,997	[15,26,32,79]	30.6 [2.0, 59.1]
Can mother transmit HCV to infants?	Mozambique, Ethiopia	Pregnant women, medicine and health science students	1,594	[26,32]	52.8 [-9.6, 115.4]
Are mosquito bites a possible route of HCV transmission?	Mozambique	Pregnant women	1,186	[26]	10.3 [8.7, 11.8]
Can Hepatitis C be transmitted through -oral route?	Ethiopia	medicine and health science students	408	[32]	70.3 [65.9, 74.6]
Is sharing toothbrush a possible route of HCV transmission?	Mozambique, Egypt	Pregnant women, HCV patients	1,389	[32,79]	52.4 [-7.3, 112.2]
Do you know any of HCV disease symptoms?	Egypt	Residents	67	[74]	55.0 [43.2, 66.7]
Can HCV infection can be asymptomatic?	Ethiopia	medicine and health science students	408	[32]	55.0 [50.2, 59.7]
Would HCV infection be prevented by vaccination?	Ethiopia, Egypt	medicine and health science students, HCV patients	611	[32,79]	42.0 [8.7, 75.3]
Can HCV be prevented by avoiding multi-sexual partnership?	Ethiopia	medicine and health science students	408	[32]	87.0 [83.8, 90.1]

#### Awareness of HCV related to demographic characteristics

Adoba and colleagues conducted their study among barbers—sharp objects-related career, nevertheless, the radio was the major source of information about HCV infection (25.0%) [15].

Demsiss and colleagues in 2018 conducted a study among medicine and health science students in Ethiopia and determined that student’s residence as well as department significantly associates to level of knowledge toward transmission and prevention of hepatitis B and C infections [32].

### Human papillomavirus (HPV)

Nineincluded studies assessed the awareness of 5,157 Africans in regard to HPV, three studies were conducted in Nigeria [35,36,41] and one in each of Madagascar, Morocco, Mali, South Africa and Senegal [43,49,51,54,70,71]. The oldest among the study included was conducted in 2010 while the newest was conducted in 2016 (Table 1). Population under study was found to be mainly adolescents and students (6/9), while two studies was targeting general population and one was targeting HIV positive and negative females (Table 1). Majority of studies were conducted among both genders (6/9), while three studies were toward females only. Age of respondents ranges from 15 to older than 67 years. Fifteen questions were asked to the participants that are related to the knowledge and awareness of HPV as general, transmission routes, clinical symptoms, pathological consequences and prevention attitude, among which 13 questions were analyzed and synthesized. The question ‘‘Do you know HPV?” was answered by 5,076 participants in Nigeria, Senegal, Morocco and Madagascar; 25.1% [95% Cl; 13.3, 37.0] answered yes. The question ‘‘Are you aware of a vaccine for the prevention of HPV?” was answered by 2,548 participants in Nigeria and Morocco; 26.1% [95% Cl; 13.3, 38.9] answered yes. Furthermore; the question ‘‘Do you know that HPV is a sexually transmitted infection” was answered by 1,409 participants in Nigeria, South Africa and Mali; 38.1% [95% Cl; 15.1, 61.2] answered yes. Questions asked, their corresponding articles’ data, the pooled prevalence and the confidence intervals are depicted in (Table 8). Heterogeneity was high in all questions (I^2^ more than 80%).

**Table 8:** Awareness of HPV related knowledge among Africans.

Question	Country/ies	Study population/s	Total sample size	References	Pooled prevalence of yes response [95% Cl]
Do you know HPV?	Nigeria; Senegal, Morocco, Madagascar	university staff and general population, mothers of female adolescents, students, Adolescents and young adults, parents	5,076	[35,36,41,49,51,54,71]	25.1 [13.3, 37.0]
Do you know that HPV can cause cervical cancer?	Nigeria, Mali	University students,university staff and general population,mothers of female adolescents,adults and adolescents	1,379	[35,36,41,70]	43.8 [19.3, 68.3]
Is HPV usually does needs no treatment?	South Africa	HIV positive and negative females	30	[43]	97.0 [90.9, 103.0]
Is Cervical cancer caused by persistent HPV infection?	Nigeria	students	368	[49]	19.6 [15.6, 23.5]
Could a person be HPV infected without knowing it?	South Africa	HIV positive and negative females, university students	310	[41,43]	79.9 [61.3, 98.5]
Does Vaccine expose adolescents to risky sexual behaviors?	Nigeria	mothers of female adolescents, university students	570	[36,41]	34.6 [-20.8, 90.1]
Does having many sexual partners increases the risk of HPV?	South Africa, Nigeria	HIV positive and negative females, students	678	[41,43,49]	65.6 [40.1, 91.1]
Do you know that HPV is a sexually transmitted infection?	South Africa, Nigeria	University students,university staff and general population, mothers of female adolescents,HIV positive and negative females, adults and adolescents	1,409	[35,36,41,43,70]	38.1 [15.1, 61.2]
Are you aware of a vaccine for the prevention of HPV?	Morocco, Nigeria	University students,HIV positive and negative females, mothers of female adolescents, students, parents	2,548	[35,36,41,49,54]	26.1 [13.3, 38.9]
Do you don’t know where or how to access HPV vaccine?	Nigeria	mothers of female adolescents, university students	570	[36,41]	53.0 [48.9, 57.0]
Is THPV vaccine too costly?	Nigeria, South Africa	mothers of female adolescents,HIV positive and negative females,university students	600	[36,41,43]	38.5 [13.6, 63.4]
Are you willing to receive the HPV vaccine?	Senegal, Mali, Nigeria	students, Adolescents and young adults	2,985	[41,49,51,70]	58.5 [9.6, 107.4]

#### Awareness of HPV related to demographic characteristics

Funmilayo and colleagues in their study detected a statistically significant association between level of awareness and vaccine acceptance as well as the level or class of students[41]. Supporting this finding; Makwe and colleagues indicated the same association[49].

Massey and colleagues in Senegal reported that respondents who indicated living most of their lives in a rural area demonstrated a greater percentage of ever having heard of HPV, and that fathers’ education level is significantly associated with the willingness of HPV vaccination. Mouallif and colleagues in Morocco concluded that mothers who agreed with the statement ‘Whatever happens to my health is God’s will’, believed that the vaccine was expensive and believed that they had insufficient information about the vaccine were significantly less likely to accept the vaccine[51,54].

### Sexually transmitted infections (STIs)

Sevenincluded studies assessed the awareness of 2,986 Africans in regard to STIs as general, three studies were conducted in Nigeria [17,18,44] and one in each of Madagascar, Morocco, Mali and Uganda [47,56,70,71]. The oldest among the study included was conducted after 2010 while the newest was conducted in 2014. Population under study was found to be mainly adolescents and students (5/7), while one study was targeting seafarers and another targeting women in reproductive age (Table 1). Majority of studies were conducted among both genders (5/7), one was toward males only while another was targeting females only (Table 1). Age of respondents range from 14 to older than 45 years. Thirty five questions were asked to the participants that are related to the knowledge and awareness of STIs general knowledge, transmission routes, clinical symptoms, pathological consequences and prevention attitude, among which 14 questions were analyzed and synthesized. The question ‘‘Is Genital ulcer a symptom of having STIs?” was answered by 2,322 participants in Morocco and Uganda; 23.5% [95% Cl; 3.8, 43.2] answered yes. The question ‘‘Do you know gonorrhea?” was answered by 1,123 participants in Nigeria and Madagascar; 22.8% [95% Cl; 5.1, 40.5] answered yes. questions asked, their corresponding articles’ data, the pooled prevalence and the confidence intervals are depicted in (Table 9). Heterogeneity was high in all questions (I^2^ more than 80%).

**Table 9:** Awareness of STIs related knowledge among Africans.

Question	Country/ies	Study population/s	Total sample size	References	Pooled prevalence of yes response [95% Cl]
Have you heard about STDs?	Nigeria	Adolescents, Students	881	[17,18]	94.5 [89.6, 99.4]
Do you know gonorrhea?	Nigeria, Madagascar	Adolescents, Students, university students	1,123	[17,18,71]	22.8 [5.1, 40.5]
Do you know syphilis?	Nigeria, Madagascar	Students, university students	782	[18,71]	5.6 [4.3, 7.0]
Do you know Herpes simplex?	Nigeria, Madagascar	Students, university students	782	[18,71]	4.7 [1.1, 8.2]
Do you know chlamydia?	Madagascar	university students	242	[71]	1.7 [0.1, 3.2]
Can Gonorrhea cause infertility?	Nigeria	students	30	[44]	60.0 [42.5, 77.4]
Is unprotected sex a mode of transmission of STIs?	Nigeria	students	540	[18]	87.0 [84.2, 89.7]
Are needles and syringes a mode of transmission OF STIs?	Nigeria	students	540	[18]	82.6 [79.4, 85.7]
Is blood and blood products a mode of transmission?	Nigeria	students	540	[18]	73.0 [69.3, 76.8]
Is Mother to child a mode of transmission of STIs?	Nigeria	students	540	[18]	70.9 [67.1, 74.6]
Is Coughing/sneezing a mode of transmission of STIs?	Nigeria	students	540	[18]	22.0 [18.6, 25.3]
Is Sharing plates a mode of transmission of STIs?	Nigeria	students	540	[18]	12.2 [9.4, 14.2]
Can Gonorrhea be transmitted to neonates?	Nigeria	students	30	[44]	21.8 [6.5, 37.0]
Is weight loss a symptom of having STIs?	Nigeria, Uganda	Students, women of reproductive age	875	[18,56]	41.4 [-28.2, 111.1]
Is Painful micturition a symptom of having STIs?	Nigeria	students	540	[18]	68.9 [65.1, 72.6]
Is Genital ulcer a symptom of having STIs?	Nigeria, Uganda, Morocco	Students, women of reproductive age, seafarers	2,322	[18,47,56]	23.5 [3.8, 43.2]
Is Genital swelling a symptom of having STIs?	Nigeria	students	540	[18]	38.4 [44.1, 52.4]
Is Genital discharge a symptom of having STIs?	Nigeria, Uganda, Morocco	Students, women of reproductive age, seafarers	2,322	[18,47,56]	34.9 [-0.9, 70.9]
Is Micturition burns a symptom of STIs in females?	Morocco	seafarers	1,447	[47]	6.2 [5.0, 7.3]
Is Micturition burns a symptom of STIs in males?	Morocco	seafarers	1,447	[47]	30.2 [27.7, 32.4]
Is Tumefaction of the groin a symptom of STIs in females?	Morocco	seafarers	1,447	[47]	1.2 [0.8, 1.5]
Is Tumefaction of the groin a symptom of STIs in males?	Morocco	seafarers	1,447	[47]	35.0 [32.6, 37.3]
Is Genital itching a symptom of STIs in females?	Morocco, Uganda	seafarers, women of reproductive age	1,782	[47,56]	34.0 [-16.2, 84.7]
Is Genital rash a symptom of STIs?	Uganda	women of reproductive age	335	[56]	14.5 [10.7, 18.2]
Is painful sex a symptom of STIs?	Uganda	women of reproductive age	335	[56]	0.3 [-1.4, 2.0]
Can Gonorrhea be Asymptomatic?	Nigeria	students	30	[44]	20.0 [5.5, 34.5]
Is abstinence a possible way to prevent STDs?	Nigeria, Uganda	Adolescents, women of reproductive age	676	[17,56]	23.2 [-19.2, 85.6]
Is condom use a possible way to prevent STDs?	Nigeria, Uganda	Adolescents, women of reproductive age	676	[17,56]	36.9 [13.4, 60.4]
Being faithful by having one sexual partner is a way to prevent STIs?	Uganda	women of reproductive age	335	[56]	26.3 [21.5, 31.0]
Do you use condoms?	Nigeria	students	30	[44]	72.5 [56.6, 88.3]
Do you reuse condoms?	Nigeria	students	30	[44]	10.1 [-0.6, 20.8]

#### Awareness of STIs related to demographic characteristics

Akokuwebe and colleagues reported that Media (as general) was the main source of information 57.0% followed by friends 30.0%, and association between source of information about STDs is significantly related to age. Moreover, Laraqui and colleagues concluded that during the year prior to the study, 73.2% of participants (seafarers) were informed about the prevention of STI/HIV/AIDS through different ways, mainly the media (73% via TV and 45.6% via radio). Amu and colleagues provided more specific information in regard to source of knowledge as they determined that there are three major sources of information; the radio and television 343 (68.7%); teachers 340 (68.1%); and newspapers 224 (44.9%). Nevertheless, Nawagi and colleagues in their study in Uganda determined that only (23.9%) of the participants have information about STIs from the media [17,47,56].

Joda and his colleagues in Nigeria conducted a study to assess the level of knowledge of STIs among students from different schools and concluded that there is no statistically significant differences in the responses obtained from various schools. Moreover, Reuter and colleagues conducted a study to assess the difference of STIs related knowledge between university students of Madagascar and USA, and concluded that there is no statistically significant differences [44,71].

In spite of the study populations’ differences, five studies reported a significant association between knowledge of STIs and the level of education [30,38,68,81,82]. Considering age as a factor influencing level of awareness; four studies report it to be significantly valid[38,50,65,81], while two studies appose [26,59]. Living in an urban area was found to be significantly associated with awareness level in several studies [38,68,83].

## Discussion

The current study was the first of its kind—to our knowledge, as not general assessment of knowledge is studied, but the specific awareness determinants. The presented outcomes are believed to be the best inputs for organizing effective preventive measures,planning and conducting awareness raising campaignsas well as identifying potential research gaps.

The current study highlights the specific levels of STIs-related knowledge, practices and prevention attitudes among different African populations. The pooled prevalence estimates showed that even though more than 90% of the population had heard about STIs (94.5%) in general and HIV(92.2%) in particular, (79.7%) had never heard about HCV. These results are consistent with earlier studies in Eastern Europe, Victoria, Lao People’s Democratic Republic and Iran [85–88]. Moreover, (25.1%) of the population knows HPV. However, a study conducted among adolescents and adult women in one of the developed countries (USA) reported that only18% had heard about the virus [89]. Nevertheless, the confounders among participants are to be considered when comparing the studies.

In the contrary to the expectations in regard to HIV-related signs and symptoms knowledge in such epidemic countries; this review revealed that almost only (14.4%), (17.0%) and (17.7%) of South Africans know that oral candidiasis, herpes zosters and constant diarrhea could be associated with HIV infection, respectively. Consistently, UNAIDS recently (2018) reported that less than (20.0%) of the same population consider TB to be associated with AIDS [90].

The current findings of knowledge related to vertical HIV transmission during pregnancy (57.0%), delivery (66.0%) or breastfeeding (73.0%) corroborate with other studies, although they slightly concluded higher proportions [91,92]. Furthermore, these findings are in line with the results reported in UAE and Greece. Nevertheless, in India; Pratibha Gupta and colleagues reported knowledge rates as low as (8.85%) and (23.8%) regarding the transmission during delivery and breastfeeding, respectively [93–95].

The findings clearly demonstrate that HIV preventative knowledge of South Africans are higher than that of Nigerians. For instance; using condom (64.4% versus52.6%) and having one sexual partner (83.1% versus 57.6%) are known to reduce HIV transmission by South African and Nigerian populations, respectively. Bangladeshi women were reported to have knowledge similar to South Africans. However, other studies conducted in Vietnam, Italy and USA reported higher proportions [96–99].

It has been reported that increased HIV knowledge resulted in a reduction of risky sexual behaviors among adolescents [100]. Notably, current findings revealed that adolescents in Africa were—for some extend aware of the facts associated with epidemics, transmission and prevention of HIV infection. Approximately (60.7%) believe that a healthy person can be HIV infected, similar finding was reported among Russians as well. Nevertheless, higher awareness rates were also reported in Iran and USA [101–103]. More than (50.0%) were found to be of good knowledge level about HIV transmission through Sexual intercourse (67.8%), Sharing sharp unsterilized objects (54.2%) and using intravenous needles (53.32%). This knowledge is higher when compared to Southern Brazilian adolescent’s. However, adolescents from India, USA, Lao People’s Democratic Republic and Iraq were reported to possess higher knowledge scores [86,97,104–106].

Despite the finding that most of adolescents in Africa are aware of HIV transmission routes, they still express extensive misconceptions; nearly the half believe that HIV could be contracted through mosquitoes (43.5%), toilet seats(43.7%),sharing cups/plates (33.5%) and through hugging or kissing (25.8%). Studies carried out among nursing students in Greece and among men who have sex with men in Finland illustrate similar findings as well. However, higher misconceptions rates(76.0%)for kissing and (100%) for each of sharing dishes, hot springs and mosquito bites were reported in Taiwan and Japan, respectively [95,107–109].

HIV-related stigma and discrimination persists as major obstacle to an effective HIV response in all parts of the world. Almost (37.4%) of South Africans consider stigma is a barrier to HIV testing. Generally speaking, Africans’ attitude toward HIV/AIDS patients is in need for enforcement. For example, (62.9%) would care for a relative with HIV in household, (57.1%) would buy vegetables from an HIV infected vendor, and only (44.8%) would allow a person with HIV to teach. Similar results were found to be reported in Sri Lanka. However, Janahi and colleagues in their findings reported that more than half of the adult participants (n = 1,630) in Bahrain would avoid sitting near, hugging or even shaking HIV infected people’s hand [110–112].

The findings presented in this study regarding HBV illustrate that the knowledge of Africans is moderate; (61.4%) know about the consequences of liver damage. Moreover, reusing needles(52.7%), sexual contact(42.5%) and toothbrush sharing(49.0%)were considered to be possible routs of HBV transmission. Furthermore, (72.3%) correctly believe in the existence of vaccination. A prior study conducted among Asian Americans in USA reported almostsimilar knowledgerates[113].

Regarding HCV;almost(68.8%) of Africansbelieve that transmission of HCV through reusing needles can occur. However, nearlythe half (42.0%) incorrectly believe in the existence of vaccination. Taiwanese dental students also believe that there is an effective vaccine for HCV but in low misconception rate as (15.0%)[107].

The pooled prevalence of the African knowledge regarding the association between HPV and cervical cancer was found to be mostly (43.8%), this is consistent with a study conducted in USA. Moreover, nearly (26.1%) of South Africans were aware of a vaccine for HPV prevention, lower knowledge rate (10.8%, N = 1,177) was reported in Berlin, Germany recently (2018). However, the fact that the later study was conducted among students and young adults is needed to beconsidered when comparing the results [114,115].

Implementations of educational awareness programs in schools will have its impacts in the near future. Moreover, knowledge raising campaigns at the continent level or nationally, in urban and rural regions, targeting infected or non infected individuals, applying traditional sittings or integrating new online tools are needed to be initiated for enhancing awareness and willingness for testing and for decreasing STIs transmission and discriminations.

### Strengths and limitations

The strengths of this review are that we systematically identified and included awareness estimates from 2010. Moreover, we have conducted meta-analysis to derive pooled prevalence estimates of all questions related. Furthermore, we carried out a quality assessment of the included studies based on criteria specifically developed to determine the quality of included studies.

Nevertheless, several limitations are to be considered when interpreting study results; grey literature evidence was not assessed. Moreover, African journals that are not indexed in the screened databases was not considered for inclusion as well, although all included studies are of good quality, several good studies might have be missed. Furthermore, another parameter that should be considered is that the limited number of participants in some questions can be observed for which the outcome might not be suitable to be generalized to the continent/country/population level. Lastly, the heterogeneity was high among the majority of questions analyzed and for the sake of this review the similar questions were considered exactly the same despite of the possibility of bias in the interview or the data collection process.

### Conclusion

The current study findings indicate that awareness is needed to be enforced. The differences observed among populations are highlighting the possibility for improvement by directing effort toward specific populations as well as addressing specific awareness determinants to ensure that gaps of weaknesses are filled.

## Supporting information

S1 Table PRISMA checklist. (DOCX)

S2 Table Assessment of quality of included studies. (DOCX)

S3 Table Characteristics of HIV-related included studies conducted among Nigerians. (DOCX)

S4 Table Characteristics of HIV-related included studies conducted among South Africans. (DOCX)

S5 Table Characteristics of HIV-related included studies conducted among adolescent Africans. (DOCX)
