# Supplementary material for: Knowledge gaps of STIs in Africa; Systematic review
Source: PLoS One. 2019 Sep 12;14(9):e0213224. doi: 10.1371/journal.pone.0213224 (PMC6742237; doi:10.1371/journal.pone.0213224)
Supplement: S2 Table — (DOCX) [file pone.0213224.s002.docx]

| **Study** | **Was the study obj clear?** | **Study sample clearly defined** | **Study pop specified and defined?** | **Participation rate above 70%?** | **Sitting and methods rigorous?** | **Analysis rigorous?** | **Total score** |
| --- | --- | --- | --- | --- | --- | --- | --- |
| Abdulai*et al*(12) | 1 | 1 | 1 | 0 | 1 | 1 | 5 |
| Abimanyi-Ochom*et al*(13) | 1 | 1 | 0 | 0 | 1 | 1 | 4 |
| Aderemi*et al* (14) | 1 | 1 | 1 | 0 | 1 | 1 | 5 |
| Adoba*et al* (15) | 1 | 1 | 1 | 0 | 0 | 1 | 4 |
| Ajide and Balogun (16) | 1 | 1 | 1 | 0 | 0 | 1 | 4 |
| Akokuwebe*et al* (17) | 1 | 1 | 1 | 0 | 0 | 1 | 4 |
| Amu and Adegun(18) | 1 | 1 | 1 | 1 | 0 | 1 | 5 |
| Appiah-Agyekum *et al* (19) | 1 | 0 | 1 | 0 | 1 | 1 | 4 |
| Aroke*et al* (20) | 1 | 1 | 1 | 0 | 0 | 1 | 4 |
| Asefa and Beyene(21) | 1 | 1 | 0 | 1 | 1 | 1 | 5 |
| Audet *et al(22)* | 1 | 1 | 1 | 0 | 0 | 1 | 4 |
| Azodo *et al*(23) | 1 | 1 | 1 | 1 | 0 | 1 | 5 |
| Becker *et al*(24) | 1 | 1 | 0 | 0 | 1 | 0 | 3 |
| Carlos *et al*(25) | 1 | 1 | 1 | 0 | 0 | 1 | 4 |
| Chan and Tsai(8) | 1 | 1 | 0 | 1 | 0 | 1 | 4 |
| Chaquisse*et al* (26) | 1 | 1 | 0 | 1 | 1 | 1 | 5 |
| Chard *et al* (27) | 1 | 1 | 1 | 1 | 0 | 1 | 5 |
| Cheng *et al* (28) | 1 | 1 | 1 | 0 | 0 | 1 | 4 |
| Chimoyi*et al* (29) | 1 | 0 | 1 | 0 | 0 | 1 | 3 |
| Ciampa *et al* (30) | 1 | 1 | 0 | 1 | 1 | 1 | 5 |
| Darteh*et al* (31) | 1 | 1 | 0 | 1 | 0 | 1 | 4 |
| Demsiss*et al* (32) | - | - | - | - | - | - | - |
| Elbadawi*et al* (33) | 1 | 1 | 1 | 1 | 0 | 1 | 5 |
| Engelbrecht *et al* (34) | 1 | 1 | 1 | 0 | 0 | 1 | 4 |
| Eni *et al* (35) | 1 | 1 | 0 | 0 | 1 | 1 | 4 |
| Ezenwa*et al* (36) | 1 | 1 | 1 | 1 | 0 | 1 | 5 |
| Faleye*et al* (37) | 1 | 1 | 1 | 1 | 0 | 1 | 5 |
| Faust *et al* (38) | 1 | 1 | 0 | 0 | 0 | 1 | 3 |
| Faye *et al* (39) | 1 | 1 | 1 | 1 | 1 | 1 | 6 |
| Frambo*et al* (40) | 1 | 1 | 0 | 1 | 0 | 1 | 4 |
| Funmilayo *et al* (41) | 1 | 1 | 0 | 0 | 0 | 1 | 3 |
| George *et al* (42) | 1 | 1 | 0 | 1 | 0 | 0 | 3 |
| Griffith *et al* (43) | 1 | 1 | 0 | 1 | 0 | 0 | 3 |
| *Joda etal(44)* | 1 | 1 | 0 | 1 | 0 | 0 | 3 |
| *Kiderlen etal(45)* | 1 | 1 | 1 | 0 | 0 | 1 | 4 |
| Kufa *etal (46)* | 1 | 1 | 0 | 1 | 0 | 1 | 4 |
| Laraqui *etal(47)* | 1 | 1 | 1 | 1 | 0 | 1 | 5 |
| Lawan *etal (48)* | 1 | 1 | 0 | 0 | 1 | 1 | 4 |
| Makwe *etal (49)* | 1 | 1 | 1 | 1 | 0 | 1 | 5 |
| Mason *etal (50)* | 1 | 1 | 1 | 0 | 0 | 1 | 4 |
| Massey *etal(51)* | 1 | 1 | 1 | 1 | 0 | 1 | 5 |
| Mesfin *etal (52)* | 1 | 1 | 1 | 0 | 0 | 1 | 4 |
| Mkumbo *etal (53)* | 1 | 1 | 1 | 1 | 0 | 1 | 5 |
| Mouallif *etal (54)* | 1 | 1 | 1 | 0 | 0 | 1 | 4 |
| Nabukenya *etal (55)* | 1 | 1 | 0 | 1 | 0 | 1 | 4 |
| *Nawagietal (56)* | 1 | 1 | 1 | 0 | 0 | 1 | 4 |
| Ngaira *etal (57)* | 1 | 1 | 1 | 0 | 1 | 0 | 4 |
| Noubiap*et al*(58) | 1 | 1 | 1 | 0 | 1 | 0 | 4 |
| Nubed*et al*(59) | 1 | 1 | 0 | 1 | 0 | 1 | 4 |
| Ojieabu*et al*(60) | 1 | 1 | 1 | 1 | 0 | 1 | 5 |
| Okonkwo*et al*(61) | 1 | 1 | 0 | 1 | 0 | 0 | 3 |
| Okonkwo*et al*(62) | 1 | 1 | 1 | 1 | 1 | 1 | 6 |
| Oladepo*and* Fayemi (63) | 1 | 1 | 1 | 0 | 1 | 0 | 4 |
| Omotowo*et al*(64) | 1 | 1 | 1 | 1 | 1 | 1 | 6 |
| Oppong and Oti-Boadi (65) | 1 | 1 | 1 | 0 | 0 | 0 | 3 |
| Owusu (66) | 1 | 0 | 1 | 0 | 0 | 1 | 3 |
| Oyekale (67) | 1 | 1 | 0 | 0 | 1 | 1 | 4 |
| Paintsil*et al*(68) | 1 | 1 | 1 | 0 | 0 | 1 | 4 |
| Pathmanathan*et al*(69) | 1 | 1 | 0 | 0 | 0 | 1 | 3 |
| Poole*et al* (70) | 1 | 1 | 1 | 0 | 1 | 1 | 5 |
| Reuter *et al* (71) | 1 | 0 | 1 | 0 | 1 | 1 | 4 |
| Rukundo *et al* (72) | 1 | 1 | 0 | 0 | 1 | 1 | 4 |
| Sahile*et al* (73) | 1 | 1 | 1 | 1 | 0 | 1 | 5 |
| Saleh *et al* (74) | 1 | 1 | 0 | 0 | 1 | 1 | 4 |
| Sandqvist*et al* (75) | 1 | 1 | 1 | 0 | 1 | 0 | 4 |
| Schwitters *et al* (76) | 1 | 1 | 1 | 0 | 1 | 1 | 5 |
| Seyoum and Legesse (77) | 1 | 1 | 1 | 0 | 1 | 1 | 5 |
| Shiferaw *et al* (78) | 1 | 1 | 1 | 0 | 0 | 1 | 4 |
| Sultan *et al*(79) | 1 | 1 | 1 | 0 | 0 | 1 | 4 |
| Tarekegne *et al*(80) | 1 | 1 | 1 | 1 | 1 | 1 | 6 |
| Umar and Oche(81) | 1 | 1 | 1 | 1 | 0 | 1 | 5 |
| Wagenaar *et al*(82) | 1 | 0 | 1 | 0 | 0 | 1 | 3 |
| Yaya *et al*(83) | 1 | 1 | 0 | 1 | 1 | 1 | 5 |
| Zungu *et al*(84) | 1 | 1 | 1 | 0 | 1 | 1 | 5 |
