# Supplementary material for: Knowledge gaps of STIs in Africa; Systematic review
Source: PLoS One. 2019 Sep 12;14(9):e0213224. doi: 10.1371/journal.pone.0213224 (PMC6742237; doi:10.1371/journal.pone.0213224)
Supplement: S3 Table — (DOCX) [file pone.0213224.s003.docx]

| **Study** | **Year of publication** | **Year/s of conduction** | **City/Region** | **study population/s** | **sample size** | **Gender** | **Participants' Age** |
| --- | --- | --- | --- | --- | --- | --- | --- |
| Aderemi*et al* (14) | 2013 | After 2010 | Oyo State | Students | 600 | Both | 12-19 |
| Ajide and Balogun (16) | 2018 | After 2010 | Ibadan/Oyo State | Students | 240 | Both | 15-17 |
| Akokuwebe*et al* (17) | 2016 | After 2010 | Ikeji-Arakeji/Osun State | Adolescents | 341 | Both | 14-18 |
| Amu and Adegun (18) | 2015 | After 2010 | Ekiti State | Students | 540 | Both | 10-14 |
| Azodo *et al* (23) | 2014 | 2010 | Enugu State | Dental tech students | 198 | Both | 20- ≥27 |
| Faust *et al* (38) | 2017 | 2013 | Nigeria | general population | 56 307 | Both | 15-49 |
| Lawan*etal*(48) | 2012 | 2011 | KanoState | FSW | 124 | Females | 24-28 |
| Ojieabu*et al* (60) | 2011 | 2011 | Sagamu/Ogun State | Pregnant Women | 403 | Females | 20- ≥40 |
| Oladepo and Fayemi (63) | 2011 | 2010 | Ibadan/Oyo State | Secondary students | 420 | Both | 10-19 |
| Umar and Oche (81) | 2012 | 2010 | Sokoto State | religious leaders | 158 | Male | ≥30 |
| Yaya *et al* (83) | 2018 | 2013 | Nigeria | community dwelling women | 38 948 | Females | 15-49 |
