# Supplementary material for: Knowledge gaps of STIs in Africa; Systematic review
Source: PLoS One. 2019 Sep 12;14(9):e0213224. doi: 10.1371/journal.pone.0213224 (PMC6742237; doi:10.1371/journal.pone.0213224)
Supplement: S4 Table — (DOCX) [file pone.0213224.s004.docx]

| **Study** | **Year of publication** | **Year/s of conduction** | **City/Region** | **study population/s** | **sample size** | **Gender** | **Participants' Age** |
| --- | --- | --- | --- | --- | --- | --- | --- |
| Becker *et al*(24) | 2015 | 2009-2010 | Durban/KwaZulu-Natal | General population | 2,477 | Both | N.A |
| Chard *et al* (27) | 2017 | After 2010 | South Africa | men who indicated an interest in men | 386 | Male | mean 33 |
| Chimoyi*et al* (29) | 2015 | 2013 | Johannesburg/Gauteng | General population | 1,146 | Both | N.A |
| Engelbrecht *et al* (34) | 2017 | 2012 | Moretele Sub-district/Bojanala District/North West province | home-based carers | 144 | Both | median 35 |
| Faleye*et al* (37) | 2014 | 2012-2013 | Durban/KwaZulu- Natal/South africa | Male medical circumcision clients | 394 | Male | mean 28±9 |
| George *et al* (42) | 2013 | 2010 | Northern Cape province | rural based traditional healers | 186 | Both | N.A |
| Kufa *etal* (46) | 2018 | After 2010 | Eastern Cape,WesternCape,FreeState,Gauteng State | STI service attendees | 1,054 | Both | 23-32 |
| Wagenaar *et al* (82) | 2012 | 2010 | South Africa | Men Who Have Sex with Men | 1,593 | Male | ≥18 |
| Zungu *et al*(84) | 2016 | 2012 | South Africa | medically and traditionally circumcised males | 11,086 | Male | ≥15 |
