# Supplementary material for: Knowledge gaps of STIs in Africa; Systematic review
Source: PLoS One. 2019 Sep 12;14(9):e0213224. doi: 10.1371/journal.pone.0213224 (PMC6742237; doi:10.1371/journal.pone.0213224)
Supplement: S5 Table — (DOCX) [file pone.0213224.s005.docx]

| **Sdy** | **Year of publication** | | **Year/s of conduction** | **City/Region/Country** | | **Study population/s** | **Sample size** | **Gender** | **Participants' Age** |  |  |
| --- | --- | --- | --- | --- | --- | --- | --- | --- | --- | --- | --- |
| Aderemi*et al* (14) | 2013 | After 2010 | | | Oyo State/Nigeria | Students | 600 | Both | 12-19 |  |  |
| Ajide and Balogun (16) | 2018 | After 2010 | | | Ibadan/Nigeria | Students | 240 | Both | 15-17 |  |  |
| Akokuwebe*et al* (17) | 2016 | After 2010 | | | Ikeji-Arakeji/Osun/Nigeria | Adolescents | 341 | Both | 14-18 |  |  |
| Amu and Adegun (18) | 2015 | After 2010 | | | Ado Ekiti/Nigeria | Students | 540 | Both | 10-14 |  |  |
| Appiah-Agyekum *et al*(19) | 2013 | After 2010 | | | Accra/Ghana | Students | 260 | Female | 16- ≥19 |  |  |
| Chaquisse*et al* (26) | 2018 | 2013-2014 | | | Nampula/Mozambique | Pregnant women | 1,186 | Female | median 22 |  |  |
| Ciampa *et al* (30) | 2012 | 2011 | | | Zambezia/Mozambique | prenatal care in women | 348 | Female | median 24 |  |  |
| Darteh*et al* (31) | | 2016 | 2011 | | | KwesimintsimZongo/Ghana | adolescents, general population | 902 | Both | mean 14 | |
| Nubed*et al* (59) | | 2016 | 2014 | | | Fako/South West Region/Cameroon | senior secondary school students | 464 | Both | 13–25 | |
| Oladepo and Fayemi 63) | | 2011 | 2010 | | | Ibadan South-West Local Government Area/Oyo/Nigeria | secondary students | 420 | Both | 10-19 | |
| Owusu*et al* (66) | | 2015 | After 2010 | | | Cape Coast Metropolis/Ghana | primary school children | 120 | Both | 9-13 | |
| Reuter *et al* (71) | | 2018 | 2013 | | | Antsiranana/Madagascar | university students | 242 | Both | 20-26 | |
| Rukundo *et al* (72) | | 2016 | 2014 | | | Kampala and Buikwe districts/Uganda | school students | 245 | Both | 10-19 | |
